# Phosphogypsum Processing into Blue Fluorescent Pigments Under Ultraviolet Excitation

**DOI:** 10.3390/molecules31132202

**Published:** 2026-06-23

**Authors:** Marina A. Egorova, Darya V. Yakhonova, Vera A. Baranova, Oleg A. Medennikov, Valentina V. Utochnikova, Anastasia V. Orlova, Nina P. Shabelskaya, Asatullo M. Radzhabov, Alexandr V. Vyaltsev, Sergey I. Sulima

**Affiliations:** 1 Department of Ecology and Industrial Safety, Faculty of Technology, Platov South-Russian State Polytechnic University (NPI), Novocherkassk 346428, Russia; m.egorova@npi-tu.ru (M.A.E.); dasha29yahonova08@gmail.com (D.V.Y.); u.vera20@yandex.ru (V.A.B.); monomors@yandex.ru (O.A.M.); nina_shabelskaya@mail.ru (N.P.S.); rajabov.asadullo@mail.ru (A.M.R.); bgd-av@mail.ru (A.V.V.); 2 Department of Nanomaterials, Faculty of Materials Sciences, Lomonosov Moscow State University, Moscow 119991, Russia; valentina@utochnikova.ru (V.V.U.); lea.rosa.17@mail.ru (A.V.O.); 3 Center of Advanced Research, National Research University “Higher School of Economics”, Moscow 108028, Russia; 4 Department of Chemical Technologies, Faculty of Technology, Platov South-Russian State Polytechnic University (NPI), Novocherkassk 346428, Russia

**Keywords:** luminophores, phosphogypsum degradation, waste recycling, ultraviolet pigments, sustainable development goals

## Abstract

In this work, we introduce the novel possibility of producing blue fluorescent ultraviolet pigments from phosphogypsum. The obtained materials are characterized by X-ray diffraction (XRD), transmission electron microscopy, and X-ray photoelectron spectroscopy (XPS). The formation of the CaS phase in the sample during the reduction of calcium sulfate was established. Thermal treatment of phosphogypsum in the presence of a reducing agent (potato starch) under environmental isolation conditions is found to yield high-quality products with high added value. The highest luminosity is established in samples containing 0.6 mol. %, which were heat-treated under a temperature of 1100 °C for 60 min. The synthesized CaS:Cu materials are shown to emit light in the blue region of the spectrum, with an emission maximum at a wavelength of 480–490 nm. The developed technological methods open the possibility to recycle chemical industry waste, which contributes to the achievement of sustainable development goals, in particular, the goal of ensuring rational consumption and production patterns.

## 1. Introduction

Luminescence is a physical phenomenon, representing the so-called cold glow of a substance, that occurs during the conversion of absorbed energy into optical radiation [1]. According to modern concepts, the mechanism of this phenomenon is based on the transition of atoms to an excited state under the influence of an external or internal source. Then, after a certain period of time, the atoms, transitioning from the excited state to the ground state, emit photons [2].

Luminophores are classified according to several criteria: their origin, glow duration, and excitation method.

According to the nature of their origin, luminophores are divided into inorganic and organic. The most well-known inorganic compounds exhibiting luminescent properties are sulfur salts [3,4], selenium [5], tellurium [6], metal phosphates [1,7,8], silicates [9,10,11,12,13], rare earth element (REE) compounds [2,14,15], borates [16,17], oxides [18], etc. Among organic compounds, substances with chains of double-conjugated bonds (benzene and its derivatives, aromatic compounds such as anthracene, naphthalene, etc.) and some groups of dyes (fluorescein, rhodamine, etc.) predominantly exhibit luminescent properties.

Most inorganic luminophores require an activator ion introduction; lanthanide cations are usually used, such as cerium [9,10,11,16], dysprosium [8,12,14], praseodymium [14,19], neodymium [20], europium [21,22,23], and others. Compositions containing inexpensive compounds of p- [24,25] or d- [7] elements as activator ions are discussed in the literature much less frequently.

The most common methods for inorganic luminophore synthesis are solid-phase [11,12,13,22,23,26,27] and sol–gel [8,15,21,24,25,28] methods. Solid-phase reactions require processes at high temperatures (1100 to 1600 °C) and last several hours. The sol–gel method produces powders at temperatures below 1000 °C but requires resources for preparing solutions and then evaporating the solvent. In both cases, luminophore production requires the use of analytical-grade materials. One of the ways to reduce the cost of phosphors may be to obtain them from industrial waste, for example, phosphogypsum.

Phosphors can be used in a wide range of fields, for example, for the manufacture of safety signs, for marking valuable objects, and for decorating interior items. Inorganic luminescent pigments possess rich color and stability. These materials are used in the production of a variety of goods, such as toys, watches, and textiles, which require a glow-in-the-dark effect, making them visually appealing to customers. In addition, phosphors are applied in optical devices and sensors. The luminescent properties of the pigments allow for their use in biological imaging and diagnostics, where they serve as effective markers or tracers. These broad applications highlight the versatility and utility of phosphors in both practical and advanced technological fields [29]. Despite the high demand for luminescent materials, their application is limited by high costs associated with production specificities. Therefore, the development of methods for obtaining low-cost luminescent materials is an urgent task.

Phosphogypsum, a byproduct of phosphate fertilizer production, accumulates in significant quantities annually, creating an environmental burden [30,31]. Phosphogypsum occupies a leading position in terms of accumulation volume among calcium sulfate-containing industrial wastes, which creates conditions for growing scientific and practical interest in possible ways to solve the problem of its disposal. As a rule, phosphogypsum is stockpiled in designated areas, forming huge waste heaps. Currently, much research is being conducted aimed at finding options for phosphogypsum utilization [32,33,34,35]. Most commonly, the use of phosphogypsum in construction is reported [32,33]. A number of research groups are working on developing technologies to process phosphogypsum into chemical products, such as calcium fluoride [34]; there are also reports on its use as a fertilizer [35]. Despite these efforts by the scientific community, the problem of phosphogypsum disposal is far from being resolved. Finding ways for its effective valorization is a pressing contemporary challenge.

Previously, we reported [36,37] on the possibility of processing phosphogypsum into inorganic luminescent materials with yellow and orange emission colors. In these studies, we obtained materials of the CaS/CaSO_4_ system with a calcium sulfide content of up to 15%. These materials exhibited the properties of phosphors, and when irradiated with ultraviolet radiation, they shone with yellow light. We also obtained materials of the composition CaS:Mn_1−x_Pb_x_ from phosphogypsum, which radiated in the yellow–orange region of the spectrum.

The aim of this study was to obtain blue luminescent pigments through the thermal treatment of phosphogypsum, a chemical industry waste product.

## 2. Results and Discussion

**Figure 1:**
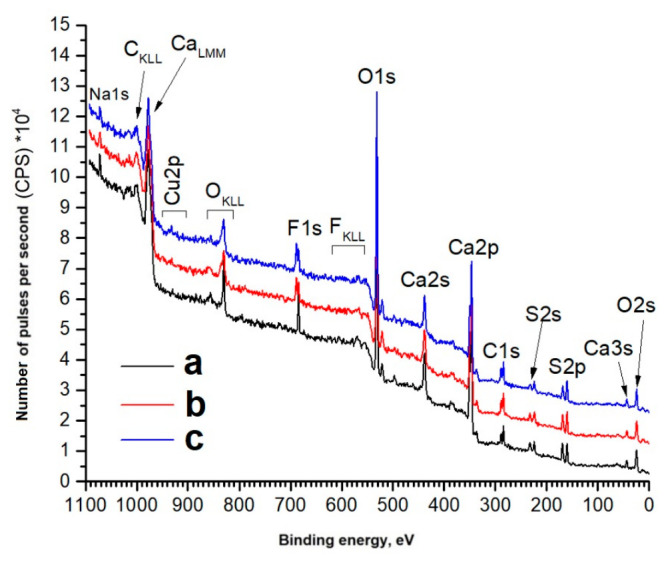
Survey spectra of phosphogypsum samples in CaS:Cu, Cu^2+^ content (mol%): a—0.4; b—0.6; c—0.8.

Survey XPS spectra of a series of samples, with the lines of the detected chemical elements indicated for a range of samples, are presented in Figure 1.

Figure 1 contains information about the elements fluorine and sodium; we assume that they could have been introduced into the sample during sample preparation.

Based on the data obtained, the concentrations of the detected elements in the surface layers of the powder samples were calculated (Table 1).

**Figure 2:**
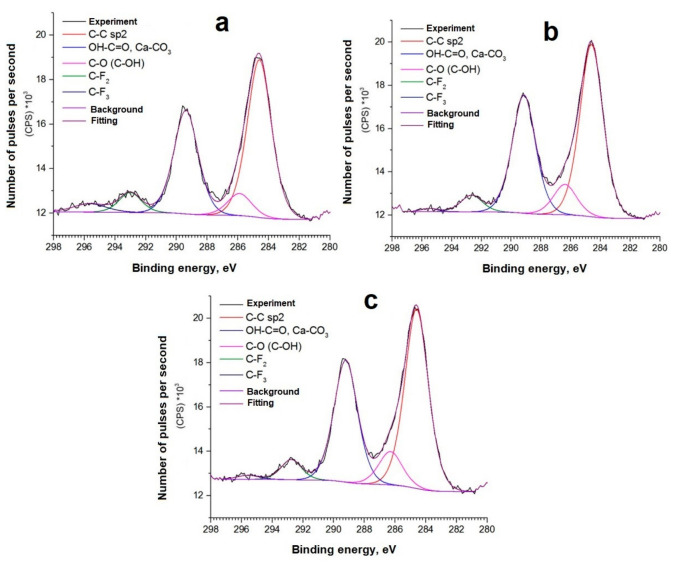
C1s spectra of CaS:Cu samples, Cu^2+^ content (mol%): (**a**)—0.4; (**b**)—0.6; (**c**)—0.8.

**Figure 3:**
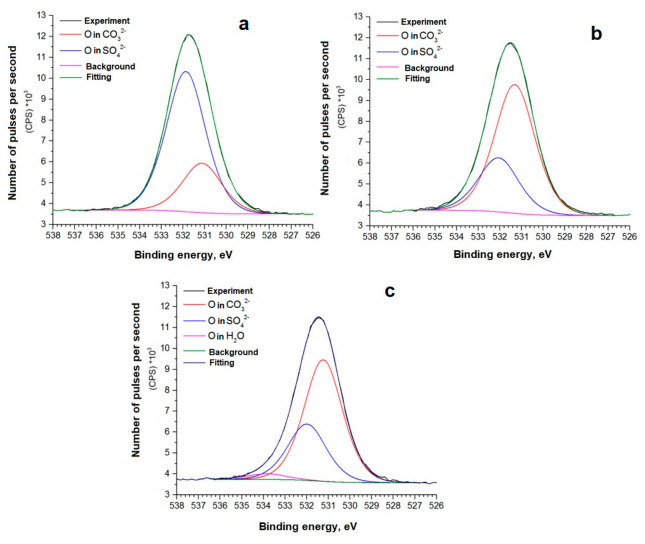
O1s spectra of CaS:Cu samples, Cu^2+^ content (mol%): (**a**)—0.4; (**b**)—0.6; (**c**)—0.8.

**Figure 4:**
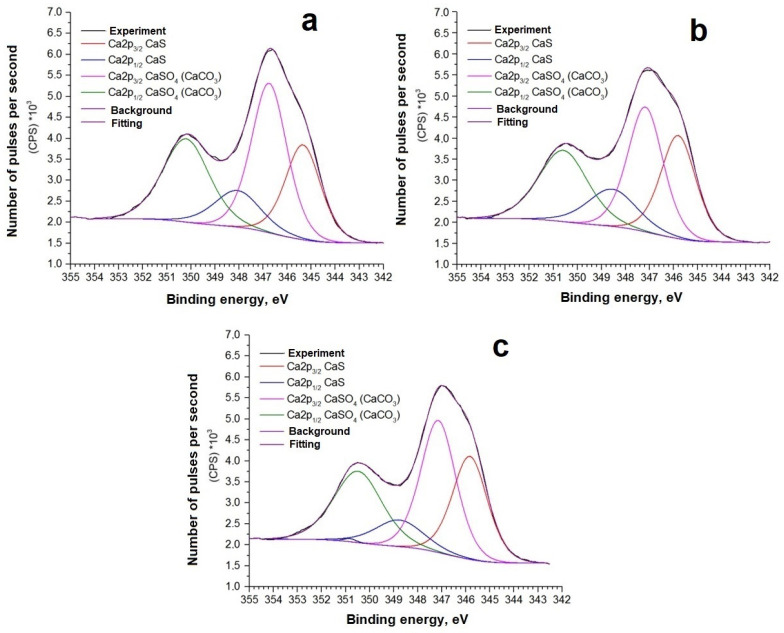
Ca2p spectra of CaS:Cu samples, Cu^2+^ content (mol%): (**a**)—0.4; (**b**)—0.6; (**c**)—0.8.

**Figure 5:**
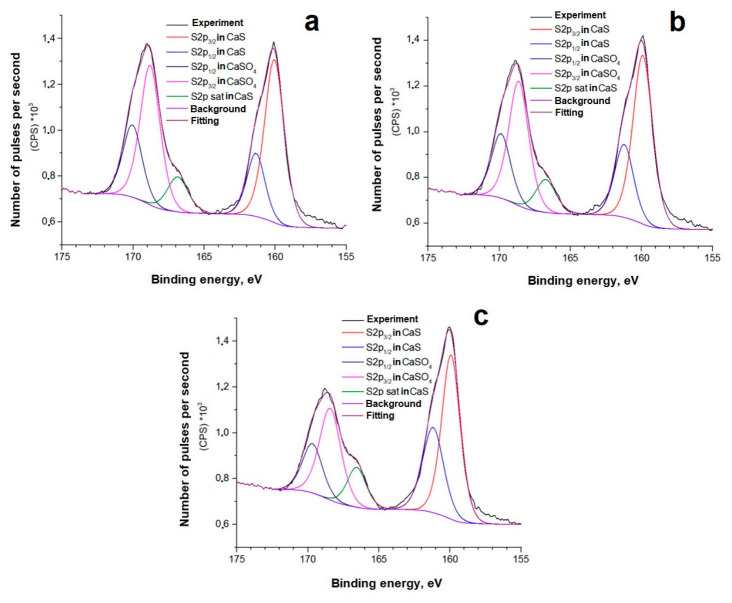
S2p spectra of CaS:Cu samples, Cu^2+^ content (mol%): (**a**)—0.4; (**b**)—0.6; (**c**)—0.8.

**Figure 6:**
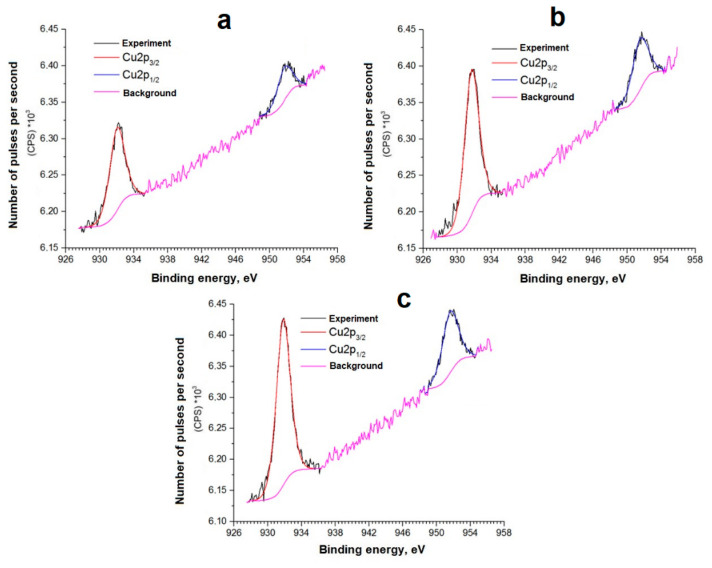
Cu2p spectra of CaS:Cu samples, Cu^2+^ content (mol%): (**a**)—0.4; (**b**)—0.6; (**c**)—0.8.

Figure 2, Figure 3, Figure 4, Figure 5 and Figure 6 below provide detailed spectra of the samples and parameters for decomposing the spectra into components, indicating the corresponding chemical bonds.

The carbon C1s spectra show a chemical bond line (Eb~289.3 eV) characteristic of CaCO_3_.

The chemical bond lines with fluorine appeared due to adsorbed contaminants from the material of the spiral pump used to pump the sample into the loading chamber and did not affect the overall XPS analysis. In the O1s spectra, with an increasing carbon content (see Table 1), the proportion of carbonate oxygen increases (Eb~531.3 eV). Additionally, oxygen bonding in SO_4_^2−^ groups is observed (Eb~532 eV).

The positions of the Ca2p and S2p lines of the samples correspond to reference values for calcium sulfide (CaS) and sulfate (CaSO_4_). The proportion of 2p sulfur bonds in sulfate relative to CaSO_4_/CaS sulfide, according to XPS data, is approximately 0.4/0.6. This quantity characterizes the phase ratio in the surface layer of the material.

The increase in peak intensity indicates an increase in Cu concentration from sample to sample, and the energy position of the Cu2p spectra indicates their charge state (2+) and their bond with sulfur.

**Figure 7:**
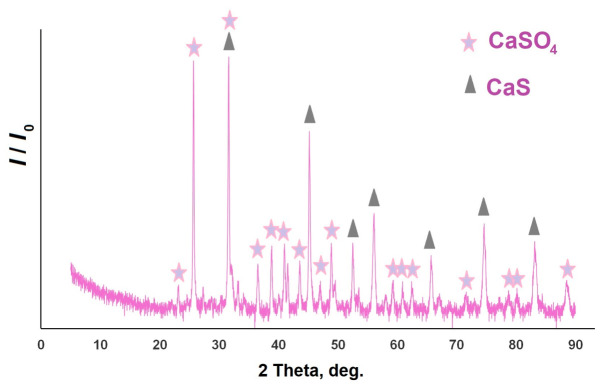
X-ray diffraction analysis of a CaS:Cu sample.

The results of the phase composition study are shown in Figure 7. According to the XRD pattern interpretation, the sample consists of calcium sulfide (PDF Number: 030-65-0894) and calcium sulfate (PDF Number: 010-70-0909). On the XRD pattern, one can distinguish peaks with 2θ values characteristic of CaSO_4_ (25.46; 31.36; 38.64; 40.82; 41.32; 43.35; 48.69; 59.02; 60.68; 62.26; 71.43; 78.62; 82.75; 88.41) and characteristic of CaS (31.36; 44.94; 53.26; 55.83; 65.44; 74.36; 82.91). Analysis of the XRD pattern confirms a CaSO_4_/CaS phase ratio of 0.4/0.6.

**Figure 8:**
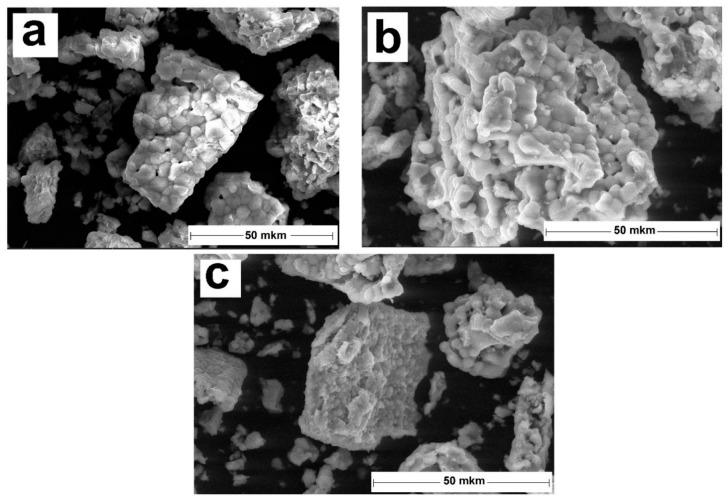
Micrographs of CaS:Cu samples, Cu^2+^ content (mol%): (**a**)—0.4; (**b**)—0.6; (**c**)—0.8.

**Figure 9:**
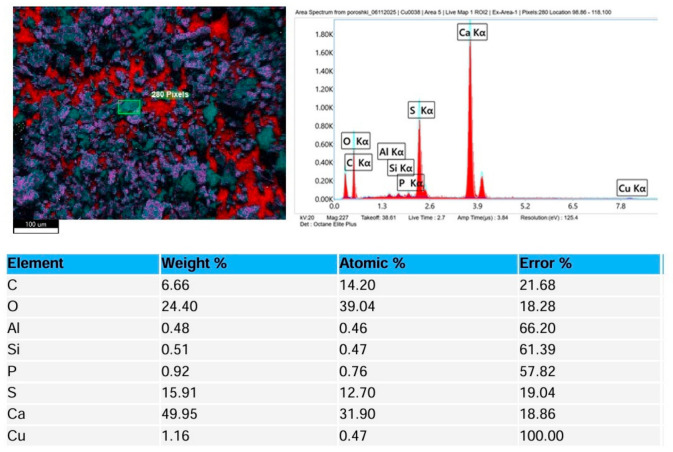
Chemical composition of a CaS:Cu sample, Cu^2+^ content 0.4 (mol%).

**Figure 10:**
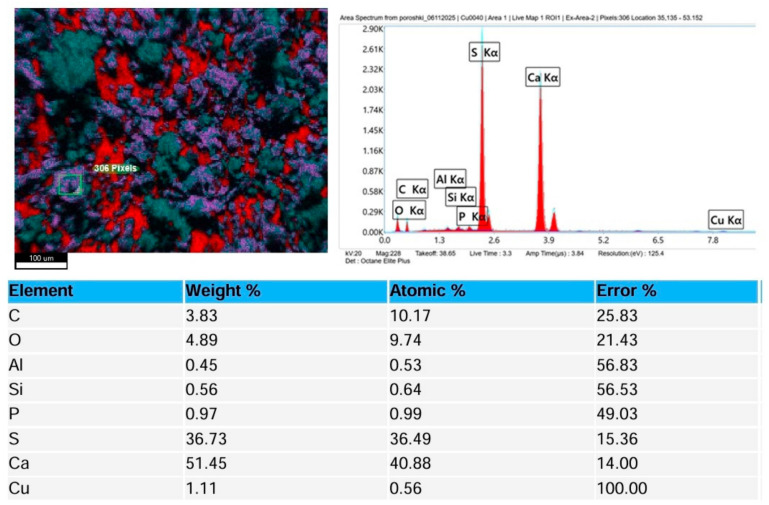
Chemical composition of a CaS:Cu sample, Cu^2+^ content 0.6 (mol%).

**Figure 11:**
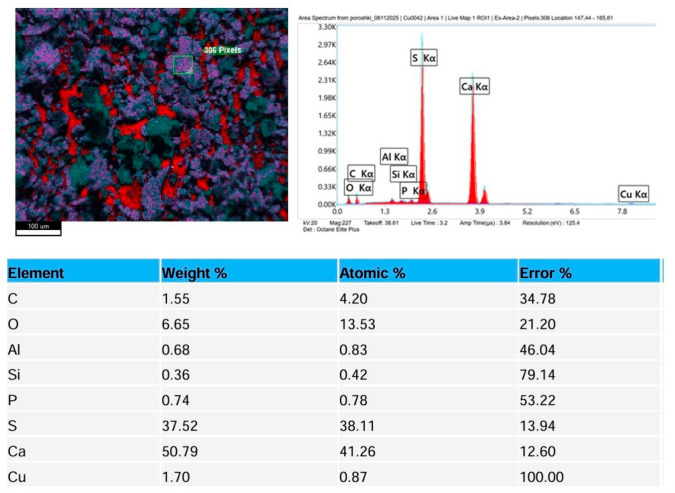
Chemical composition of a CaS:Cu sample, Cu^2+^ content 0.8 (mol%).

Figure 8 shows micrographs of CaS:Cu samples. The morphology consists of material particles ranging in size from 20 to 100 µm, grouped into assemblies of smaller particles. As a result of the reduction process, cubic sulfide crystals form on the surface of the calcium sulfate crystal, which has a lamellar structure. The results of the chemical composition elemental analysis are presented in Figure 9, Figure 10 and Figure 11 (Supplementary Files S1–S3 provide more detailed information).

During the heat treatment of phosphogypsum in the presence of a reducing agent (potato starch), reaction (1) can occur (in general form):

3CaSO_4_·2H_2_O + C_6_H_10_O_5_ → 3CaS + 6CO_2_ + 11H_2_O. (1)

The interaction processes of phosphogypsum with the reducing agent can be described by Equations (2)–(8):

C_6_H_10_O_5_ = 6C + 5H_2_O, Δ*G*_r_ → −169.06 kJ; (2)

C_6_H_10_O_5_ + 3O_2_ = 6CO + 5H_2_O, Δ*G*_r_ → −995.08 kJ; (3)

C_6_H_10_O_5_ + CaSO_4_·2H_2_O = CaS + 7H_2_O + 4CO + 2C, Δ*G*_r_ → −72.76 kJ; (4)

CaSO_4_·2H_2_O + 4CO = CaS + 2H_2_O + 4CO_2_, Δ*G*_r_ → −166.89 kJ; (5)

CaSO_4_ + 4CO = CaS + 4CO_2_, Δ*G*_r_ → −400.73 kJ; (6)

CaSO_4_ + 4C = CaS + 4CO, Δ*G*_r_ → 442.24 kJ, *t* = 432 °C; (7)

CaSO_4_ + 2C = CaS + 2CO_2_, Δ*G*_r_ → 52.93 kJ, *t* = 170 °C. (8)

Most of these processes have a negative Gibbs energy value, indicating the thermodynamic possibility of their occurrence. In reactions (7) and (8), a thermodynamic barrier exists, but it is overcome when the system is heated to a temperature above 400 °C. The possibility of calcium sulfide formation from phosphogypsum during heat treatment under a reducing atmosphere is reported, for example, in [38].

The calcium sulfide obtained as a result of the reduction serves as a matrix for CaS:Cu phosphor.

**Figure 12:**
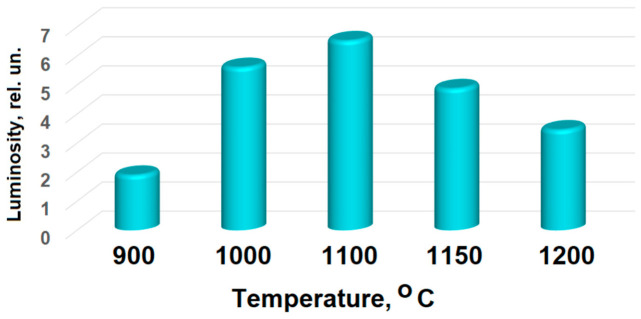
Maximum relative luminescence of samples as a function of heat-treatment temperature.

**Figure 13:**
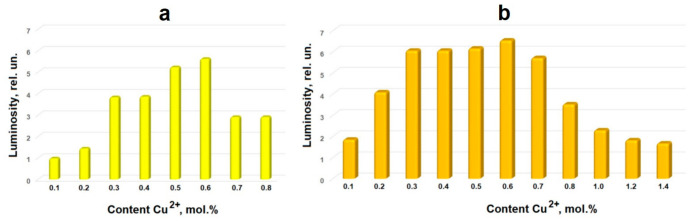
Maximum relative luminescence of samples as a function of dopant content at heat-treatment temperature of: (**a**)—1000 °C, (**b**)—1100 °C.

According to the results obtained, across all studied ranges of Cu^2+^ dopant concentrations, the most pronounced luminescence was observed for samples heat-treated at 1100 °C. The most pronounced luminescence intensity was observed with a content of 0.6% mol of copper ions (Figure 12 and Figure 13).

The effect of heat-treatment duration on the luminosity of reduced phosphogypsum samples doped with copper cations was studied.

**Figure 14:**
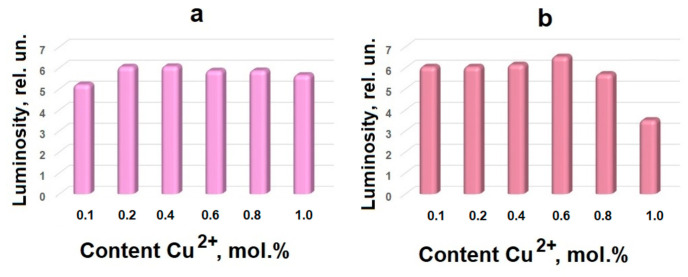
Dependence of the maximum relative luminosity of the samples on the dopant content during heat treatment at 1100 °C: (**a**)—30 min, (**b**)—60 min.

To determine the required heat-treatment time, a copper (II) salt solution (5 g Cu^2+^/L) was added to 17.2 g of phosphogypsum and dried to constant weight at 100 °C. After that, 7 g of starch was added to the samples, which were then thoroughly ground in a mortar until a homogeneous mixture was formed. Afterwards, the samples were placed in a muffle furnace, where they were heat-treated at 1100 °C. Heating was performed at a rate of 13 °C/min, with isothermal holding times of 30, 60, and 120 min. The results are presented in Figure 14.

Evidently, materials heat-treated at 1100 °C for 60 min exhibited the highest luminosity.

Thus, based on the conducted research, we conclude that the optimal process conditions for producing blue-glow ultraviolet pigment samples based on reduced phosphogypsum are as follows: heat-treatment temperature of 1100 °C, isothermal holding time of 60 min, temperature rise rate of 13 °C/min, sample cooling with a furnace; the amount of dopant added is 0.1–1.0 mol %.

It was not possible to determine the dependence of the luminescence intensity on the CaSO_4_ content in the sample. For example, in a sample with 0.6 mol. % Cu and 41.1% CaSO_4_ (average value), the luminosity was maximum.

**Figure 15:**
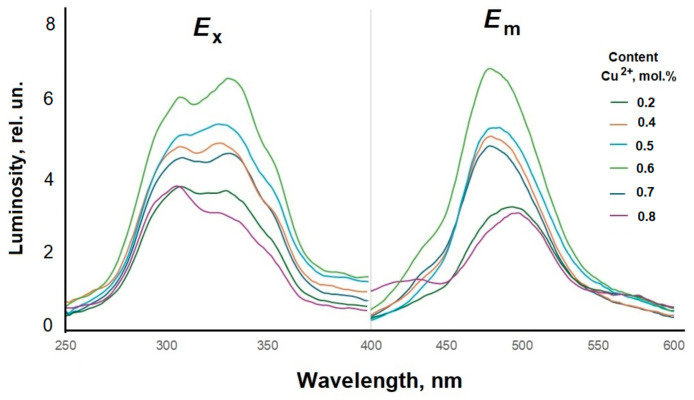
Excitation (*E*_x_) and luminescence (*E*_m_) spectra for CaS:Cu samples.

Figure 15 shows the excitation (*E*_x_) and luminescence (*E*_m_) spectra of phosphogypsum samples doped with copper cations; the amount of dopant added varies from 0.2 to 0.8 mmol.

**Figure 16:**
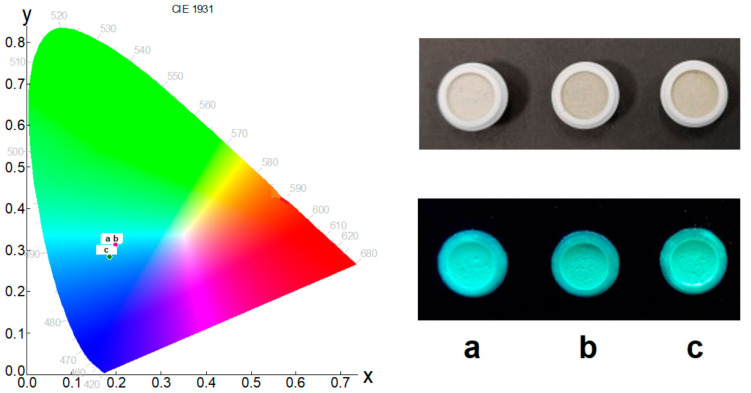
Chromaticity diagram for reduced CaS:Cu samples, Cu^2+^ content (mol%): (a)—0.4; (b)—0.6; (c)—0.8.

Figure 16 shows the luminescence chromaticity diagram. Table 2 lists the luminescence characteristics of the material: luminescence excitation maximum wavelength, luminescence peak wavelengths, average luminescence lifetime, and luminescence quantum yield.

According to the data obtained, the maximum luminescence excitation wavelength is 336–400 nm, with a peak corresponding to 483–487 nm observed in the luminescence spectrum. This produces a blue glow, which is visible on the luminescence chromaticity diagram (Figure 16). The average luminescence lifetime is ~3007 ns, and the quantum yield was 0.07–0.14. The synthesized samples exhibit the properties of phosphorescent materials (for these materials, the luminescence lifetime ranges from 10–3 s to several hours).

The synthesized materials have relatively low quantum yields (7–14%) and are inferior to classical phosphors (for example, for compounds with cerium cations CaLu_2_MgAl_2_Si_2_O_12_:Ce^3+^, a quantum yield value of 83.02% was obtained [39], and for LaLi_0.333_Ti_0.667_O_3_: 0.3 mol% Mn^4+^, this value is 47% [40]. However, the compounds that we have obtained have an undoubted advantage: they are obtained from industrial waste, without additional purification, using a simple technology. Thus, the task of being involved in the recycling of industrial waste and obtaining the required materials is achieved.

**Figure 17:**
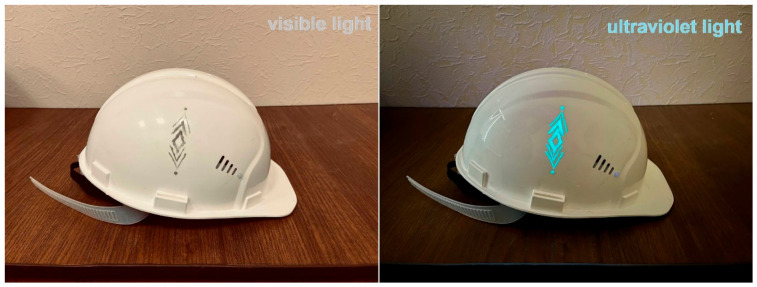
Photograph of an item decorated with the developed pigment under natural light (**left**) and under UV illumination (**right**).

Since calcium sulfide is present in the main phase of obtaining the pigments, which is unstable to moisture, the pigments should be stored in closed containers. Using the pigments synthesized, paints and varnishes were obtained. Figure 17 shows a photograph of a product decorated with the developed materials under normal lighting and under long-wavelength ultraviolet light.

Nitrocellulose lacquer was used as a base, into which 10–30% pigment was added. The lacquer matrix isolated the pigment from the surrounding environment, making it suitable for decorating objects.

## 3. Materials and Methods

### 3.1. Materials

We used agricultural-grade phosphogypsum (EuroChem-Belorechenskiye Mineral Fertilizers LLC, Belorechensk city, Russia) to obtain our phosphors. This phosphogypsum contained 99% calcium sulfate dihydrate. Copper cations were introduced in the form of a solution with a concentration of 0.08 mol/L, using CuSO_4_·5H_2_O (Lenreactive JSC, Saint Petersburg, Russia) as the starting material. Distilled water was used. Potato starch was employed to create a reducing environment in the reaction zone.

### 3.2. Synthesis

To study the effect of the amount of doping cation introduced, a series of experiments were conducted. The mass of phosphogypsum in the samples was fixed at 17.2 g. Potato starch in the amount of 7 g was used as a reducing agent.

A copper (II) salt solution containing 5 g of ions per liter (15.3 g CuSO_4_·5H_2_O) of solution was prepared.

Phosphogypsum samples were mixed with the salt solution, then placed in a drying oven and dried at 100 °C to constant mass (Drying cabinet Stegler FDO-30b, China). After that, the mixture was ground in a mortar until passing through a 0.1 mm sieve. Next, the phosphogypsum was mixed with a reducing agent and loaded into small crucibles (30–50 mL), which were then inverted and placed with the mixture into larger crucibles. A more detailed description of the method can be found in our paper [37]. The crucibles were placed in the working space of a muffle furnace, where they were heat-treated at 800–1200 °C (with a temperature rise rate of 13 °C/min (Muffle furnace SNOL 6,7/1300, manufactured by AB “Umega”, Utena, Lithuania)). The samples cooled inside the furnace along with it.

The amount of doping cations introduced varied from 0.1 to 1.4% mol.

### 3.3. Characterization

The obtained materials were studied using a number of methods. The phases within the sample were investigated on an ARL X’TRA (Thermo Fisher Scientific, Waltham, MA, USA) instrument in the 2θ angular range from 5° to 90°. Radiation with a wavelength of 1.54051 Å (copper) was used.

To obtain information on the elemental composition, a Quattro S SEM (Thermo Fisher Scientific, USA) instrument and an Octane Elite Plus (EDAX) microanalyzer (Mahwah, NJ, USA) were utilized.

The luminescent characteristics of the samples were recorded on a CM 2203 spectrofluorometer (Minsk city, Belarus).

Refinement of the elemental composition and valence of the ions was performed on a SPECS instrument (“SPECS GmbH-Surface Analysis and Computer Technology”, Berlin, Germany). The luminescent properties are investigated in the following modes: the excitation wavelength for copper samples is 330 nm, and the emission maximum is 480 nm. Non-monochromatized X-ray radiation from a magnesium anode (hv = 1253.6 eV) was used. The energy analyzer is a hemispherical deflector Phoibos-150. The samples were prepared for analysis by applying the powder to an indium substrate by “rolling out” using a glass tube.

## 4. Conclusions

Based on our comprehensive study of copper (II) cation-doped sulfide luminophores from phosphogypsum formation conditions, we can draw the following conclusions:

1. We have introduced a novel production possibility to obtain blue-emitting ultraviolet pigments from phosphogypsum. A calcium sulfide matrix is shown to form during phosphogypsum heat treatment in the presence of a reducing agent.
2. The highest luminosity is established in samples containing 0.6% mol. at a heat-treatment temperature of 1100 °C for 60 min.
3. The synthesized CaS:Cu materials emit in the blue region of the spectrum with a peak emission wavelength of 480–490 nm.
4. The results obtained open up broad possibilities for using phosphogypsum, a large-scale waste product of the chemical industry, to produce highly innovative products.

## Figures and Tables

**Table 1:** Element concentrations in the surface layers of CaS:Cu samples according to XPS data (at%).

Amount of Cu^2+^, % (mol.)	Content, at. %				
	C	O	Ca	S	Cu
0.4	17.90	45.60	22.40	14.00	0.1
0.6	19.70	45.50	21.10	13.55	0.15
0.8	20.60	44.60	21.40	13.20	0.20

**Table 2:** CaS:Cu sample luminescence characteristics.

Amount of Cu^2+^, % (mol.)	Maximum Luminescence Excitation Wavelength, λ_em,_ nm	Maximum Luminescence Wavelength, λ_em,_ nm	Luminescence Lifetime, τ_avr,_ ns	Quantum Yield, *Φ*_F_
0.4	336, 398	483	3071	0.14
0.6	336, 399	487	2897	0.07
0.8	336, 400	485	3053	0.10

## Data Availability

Data are contained within the article and Supplementary Materials.

## Supplementary Materials

The following supporting information can be downloaded at: https://www.mdpi.com/article/10.3390/molecules31132202/s1.
