# Supplementary material for: Phosphogypsum Processing into Blue Fluorescent Pigments Under Ultraviolet Excitation
Source: Molecules. 2026 Jun 23;31(13):2202. doi: 10.3390/molecules31132202 (PMC13363016; doi:10.3390/molecules31132202)
Supplement: Supplementary file 1 [file molecules-31-02202-s001.zip › S 1.pdf]

EDAX APEX

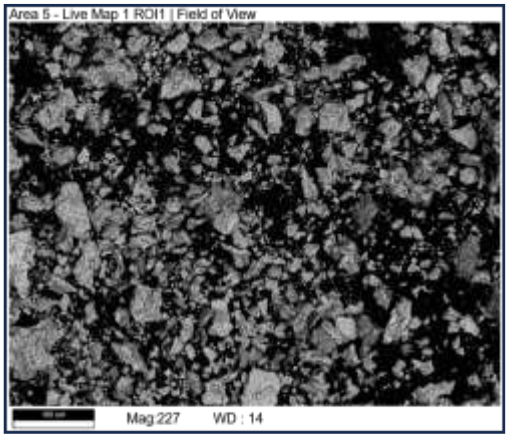

Area 5 - Live Map 1 ROI1 | Field of View
Mag:227
WD : 14

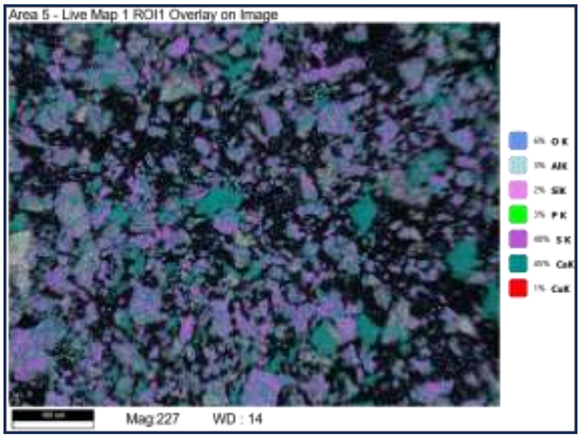

Area 5 - Live Map 1 ROI1 Overlay on Image
O K
AlK
SiK
P K
S K
CaK
CaK
Mag:227
WD : 14

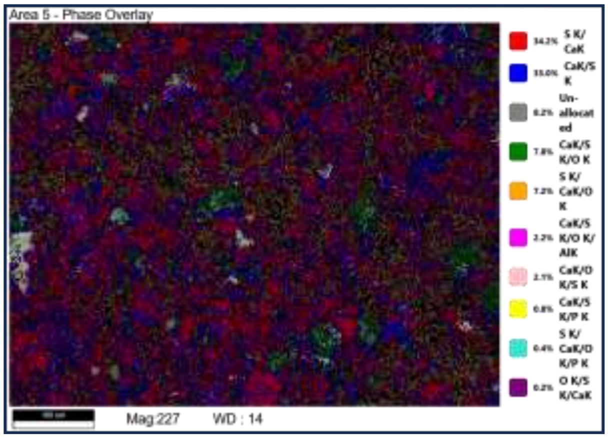

Area 5 - Phase Overlay
S K/ CaK
CaK/S K
Un-allocated
CaK/S K/O K
S K/ CaK/O K
CaK/S K/O K/ AlK
CaK/O K/S K
CaK/S K/P K
S K/ CaK/O K/P K
O K/S K/CaK
Mag:227
WD : 14

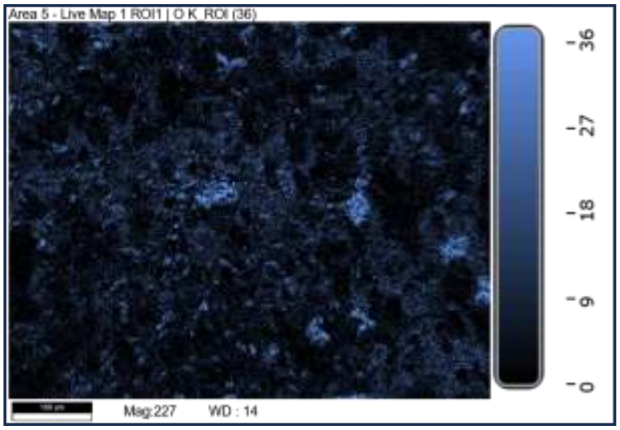

Area 5 - Live Map 1 ROI1 | O K_ROI (36)
36
27
18
9
0
Mag:227
WD : 14

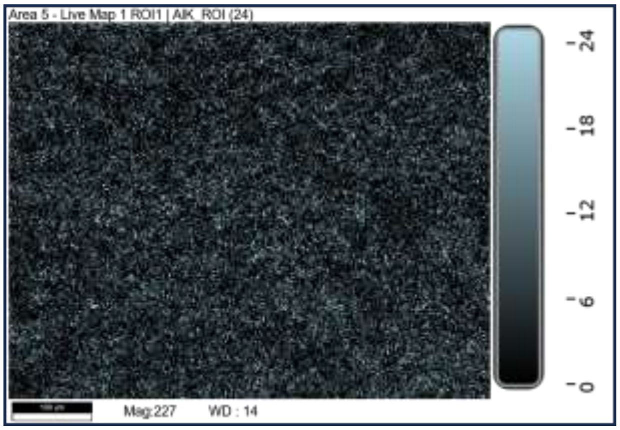

Area 5 - Live Map 1 ROI1 | AlK_ROI (24)
24
18
12
6
0
Mag:227
WD : 14

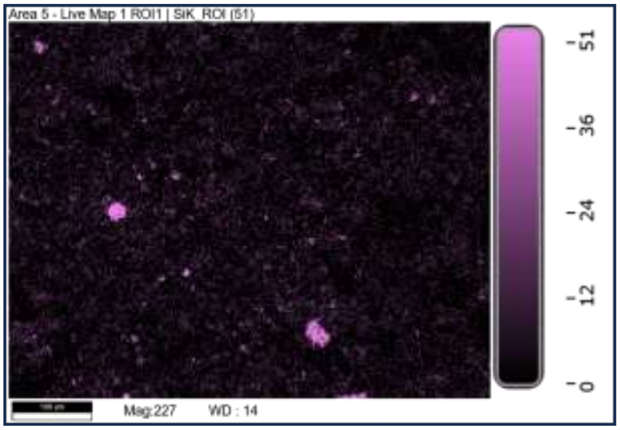

Area 5 - Live Map 1 ROI1 | SiK_ROI (51)
51
36
24
12
0
Mag:227
WD : 14

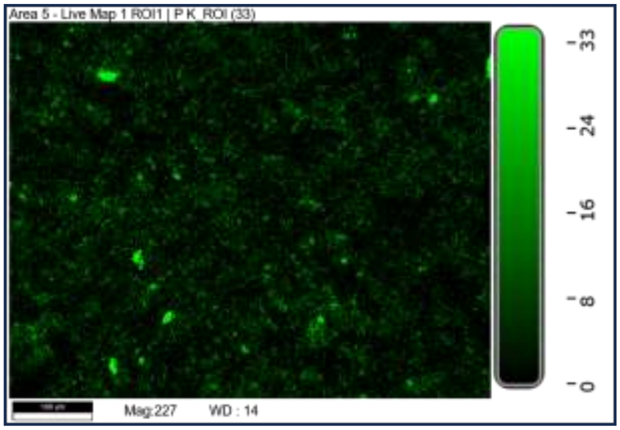
Area 5 - Live Map 1 ROI1 | P K_ROI (33)
33
24
16
8
0
Mag:227
WD : 14

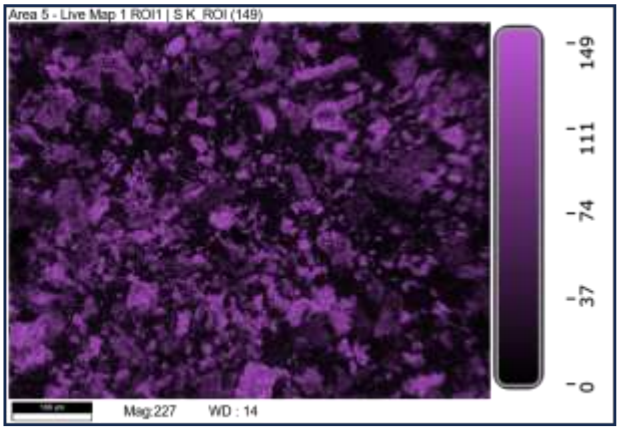
Area 5 - Live Map 1 ROI1 | S K_ROI (149)
149
111
74
37
0
Mag:227
WD : 14

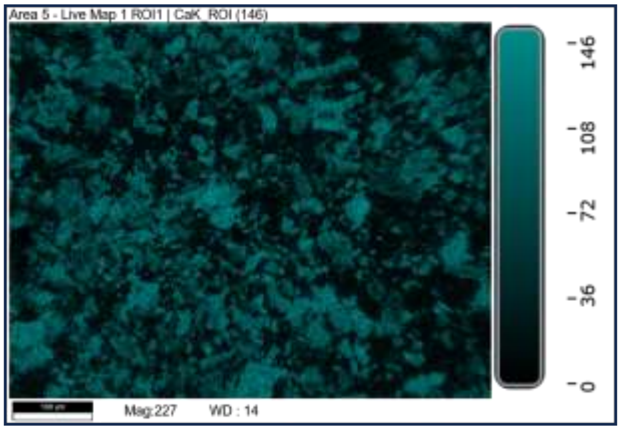
Area 5 - Live Map 1 ROI1 | CaK_ROI (146)
146
108
72
36
0
Mag:227
WD : 14

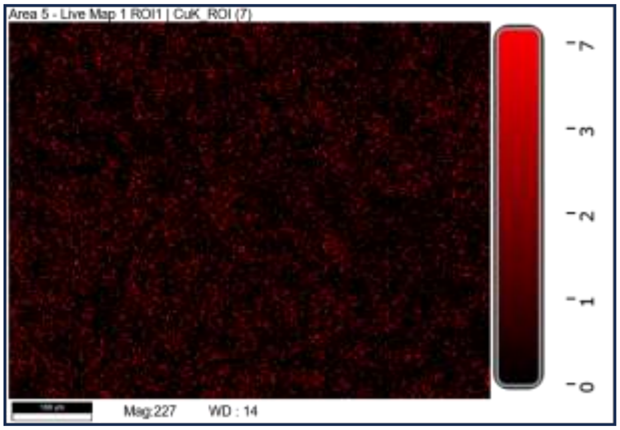
Area 5 - Live Map 1 ROI1 | CuK_ROI (7)
7
3
2
1
0
Mag:227
WD : 14

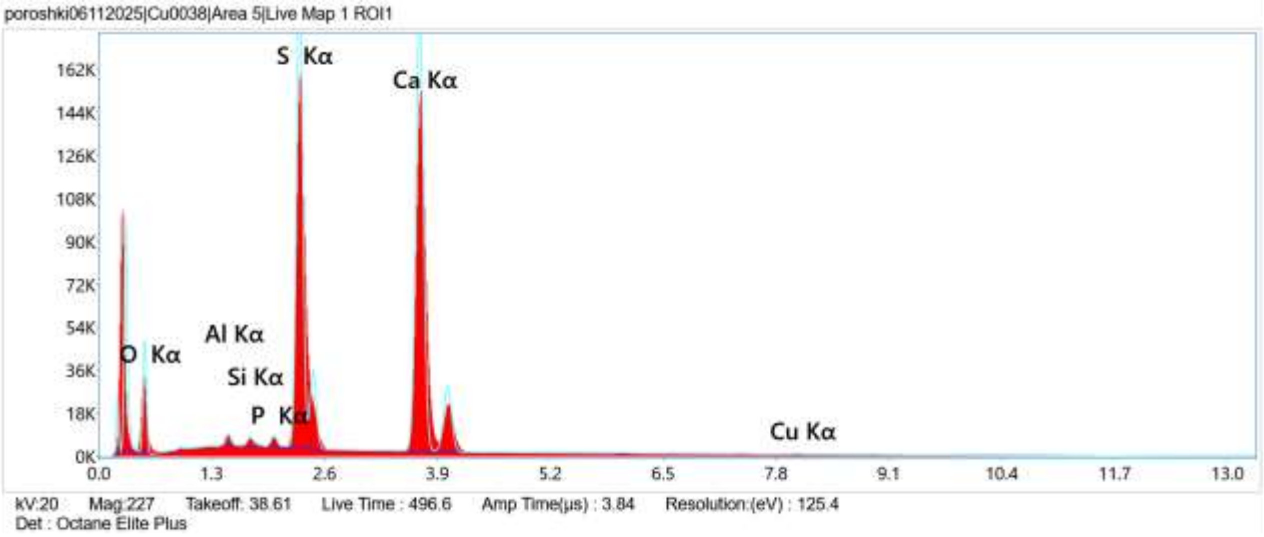

PeBaZAF Quant Result - Analysis Uncertainty: 99.00 %

Element	Weight %	Atomic %	Error %	A
O K	24.03	41.82	15.41	1.0000
Al K	0.52	0.54	7.45	1.0474
Si K	0.38	0.38	8.81	1.0359
P K	0.69	0.62	7.68	1.0270
S K	29.03	25.22	3.71	1.0205
Ca K	45.03	31.29	2.51	1.0152
Cu K	0.32	0.14	48.78	1.0053

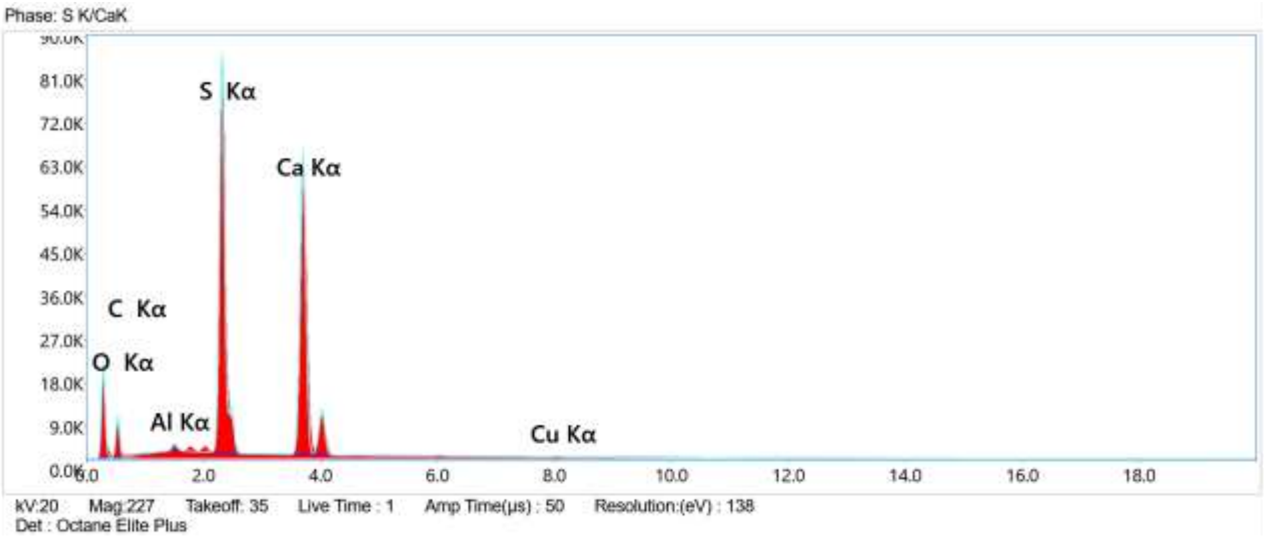

PeBaZAF Quant Result - Analysis Uncertainty: 99.00 %

Element	Weight %	Atomic %	Error %	A
C K	45.95	65.43	15.54	1.0000
O K	14.91	15.93	15.76	1.0000
Al K	0.32	0.20	9.42	1.0470
S K	17.84	9.52	3.37	1.0194
Ca K	20.75	8.85	2.77	1.0130
Cu K	0.22	0.06	44.16	1.0036

EDAX APEX

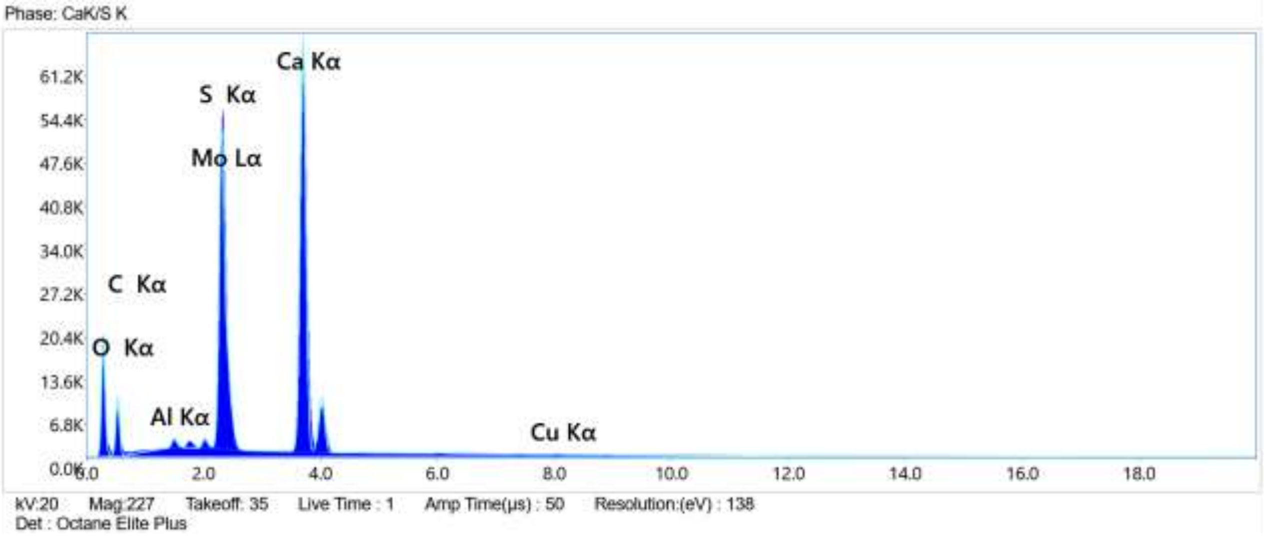

PeBaZAF Quant Result - Analysis Uncertainty: 99.00 %

Element	Weight %	Atomic %	Error %	A
C K	30.87	52.14	15.57	1.0000
O K	20.77	26.33	15.74	1.0000
Al K	0.45	0.34	8.71	1.0540
S K	7.24	4.58	4.11	1.0237
Ca K	27.13	13.73	3.03	1.0179
Cu K	0.25	0.08	57.91	1.0049
Mo L	13.28	2.81	3.98	1.0240

EDAX APEX

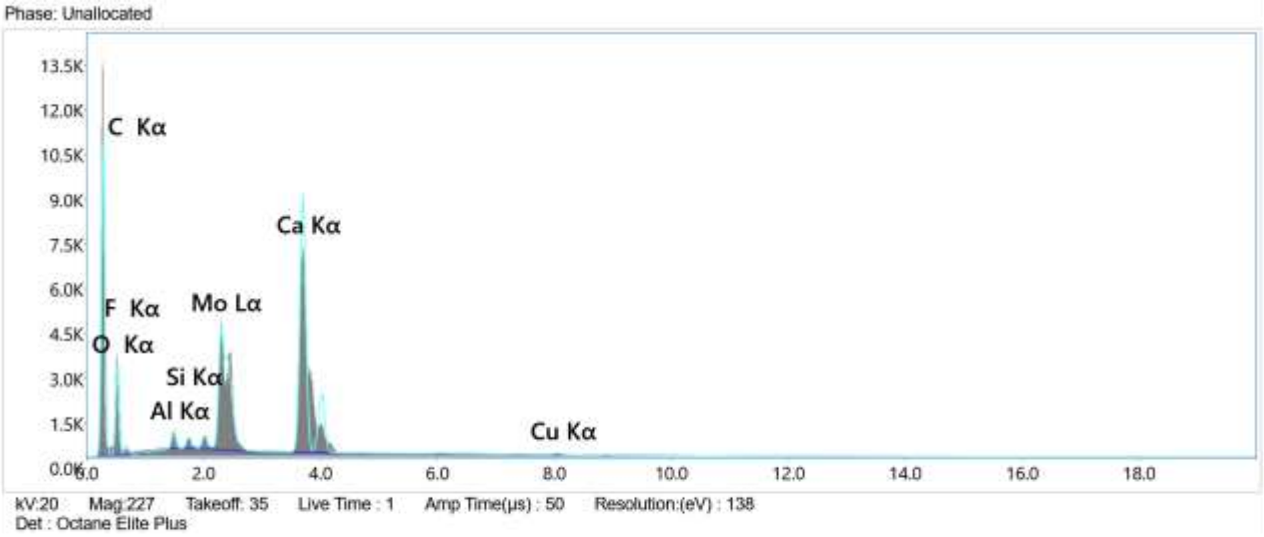

PeBaZAF Quant Result - Analysis Uncertainty: 99.00 %

Element	Weight %	Atomic %	Error %	A
C K	42.19	61.22	15.72	1.0000
O K	24.24	26.40	16.31	1.0000
F K	1.98	1.82	18.95	1.0000
Al K	0.66	0.42	12.94	1.0533
Si K	0.42	0.26	18.70	1.0412
Ca K	16.99	7.39	5.19	1.0154
Cu K	0.39	0.11	74.84	1.0037
Mo L	13.15	2.39	5.86	1.0240

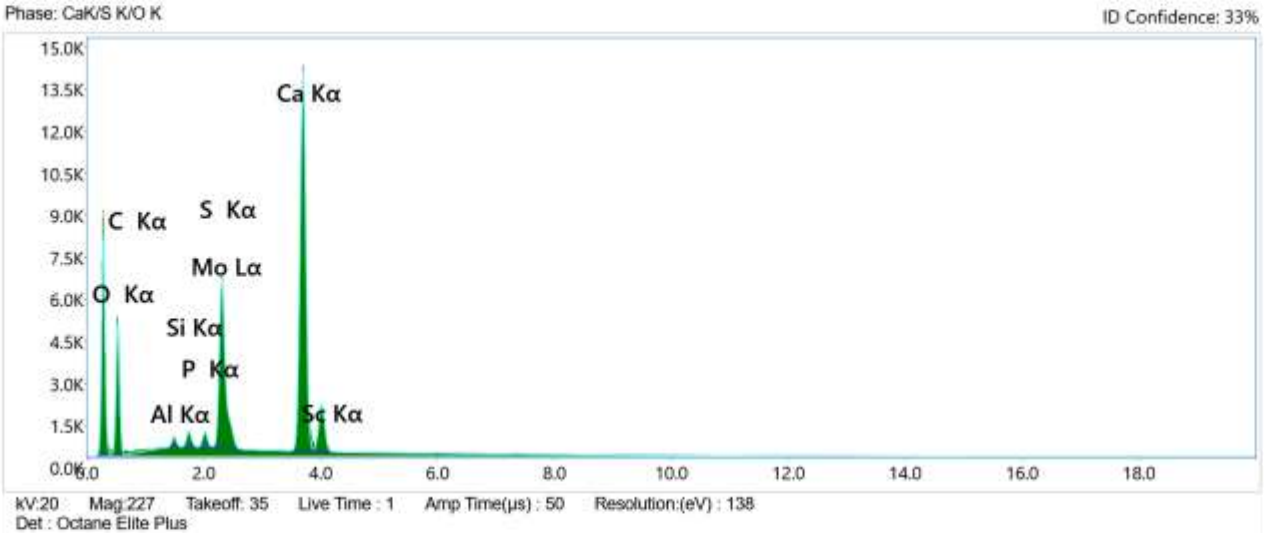

PeBaZAF Quant Result - Analysis Uncertainty: 99.00 %

Element	Weight %	Atomic %	Error %	A
C K	61.90	73.21	15.61	1.0000
O K	23.72	21.06	16.13	1.0000
Al K	0.36	0.19	14.73	1.0456
Si K	0.17	0.09	27.74	1.0343
P K	0.24	0.11	23.09	1.0255
S K	5.93	2.63	5.11	1.0191
Ca K	7.68	2.72	5.75	1.0084

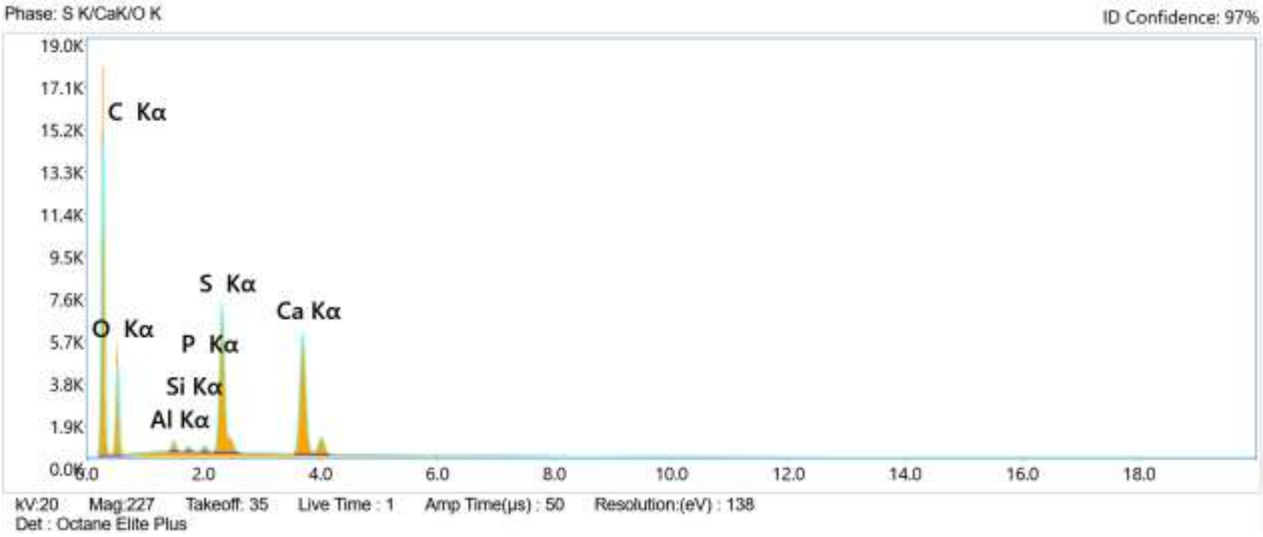

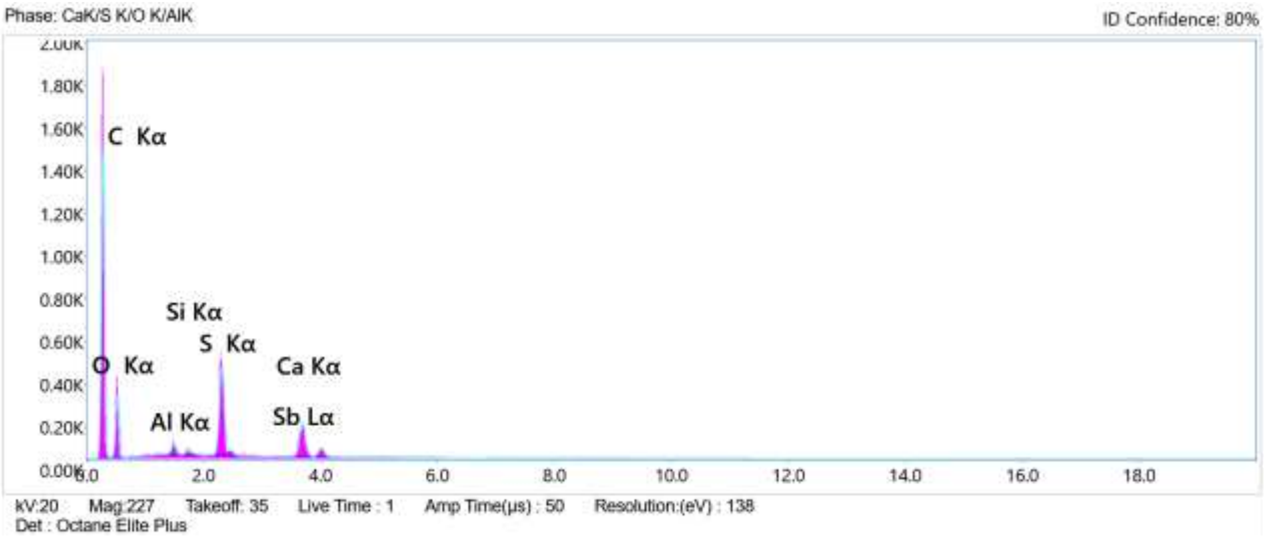

PeBaZAF Quant Result - Analysis Uncertainty: 99.00 %

Element	Weight %	Atomic %	Error %	A
C K	68.29	79.11	9.65	1.0000
O K	17.20	14.96	27.58	1.0000
Al K	0.87	0.45	40.94	1.0433
Si K	0.41	0.20	85.56	1.0329
S K	8.02	3.48	16.48	1.0182
Ca K	5.21	1.81	20.38	1.0089

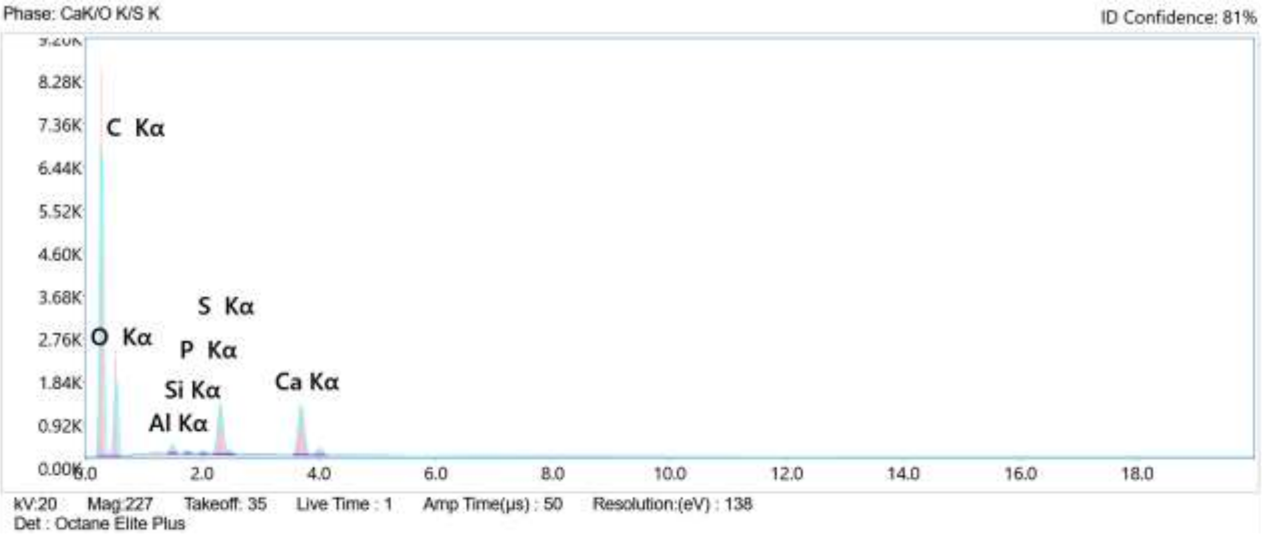

PeBaZAF Quant Result - Analysis Uncertainty: 99.00 %

Element	Weight %	Atomic %	Error %	A
C K	61.84	73.97	10.36	1.0000
O K	21.44	19.25	24.92	1.0000
Al K	0.93	0.49	21.76	1.0453
Si K	0.31	0.16	55.54	1.0347
P K	0.40	0.19	51.62	1.0260
S K	6.00	2.69	10.12	1.0196
Ca K	9.09	3.26	11.09	1.0086

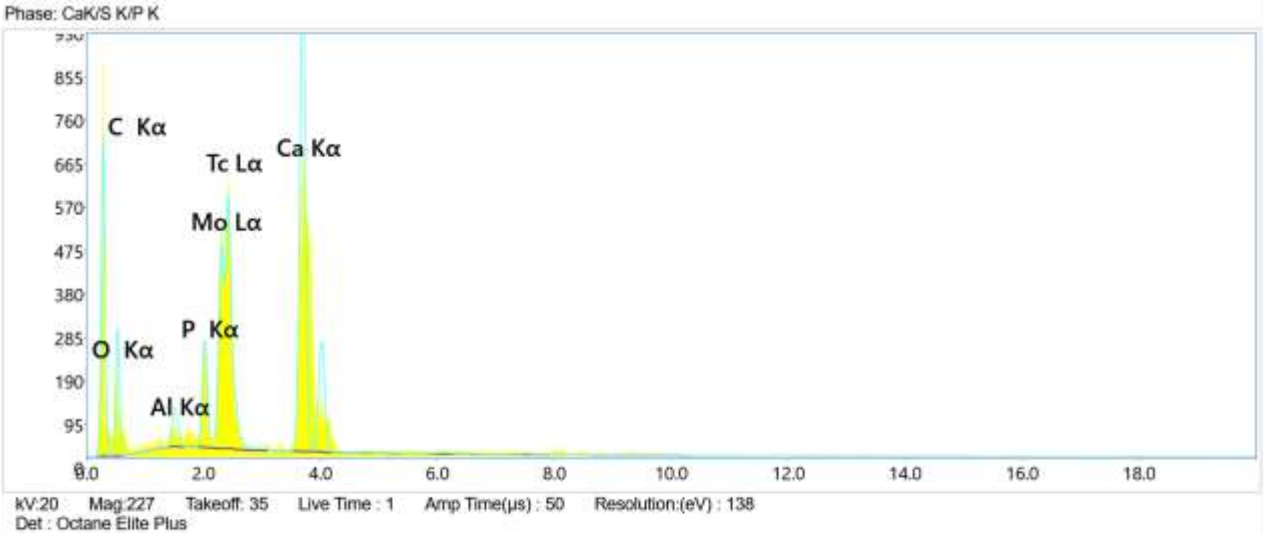

PeBaZAF Quant Result - Analysis Uncertainty: 99.00 %

Element	Weight %	Atomic %	Error %	A
C K	63.15	74.37	16.17	1.0000
O K	22.64	20.01	17.40	1.0000
Al K	1.05	0.55	16.01	1.0455
S K	4.74	2.09	9.54	1.0191
Ca K	8.42	2.97	10.54	1.0079

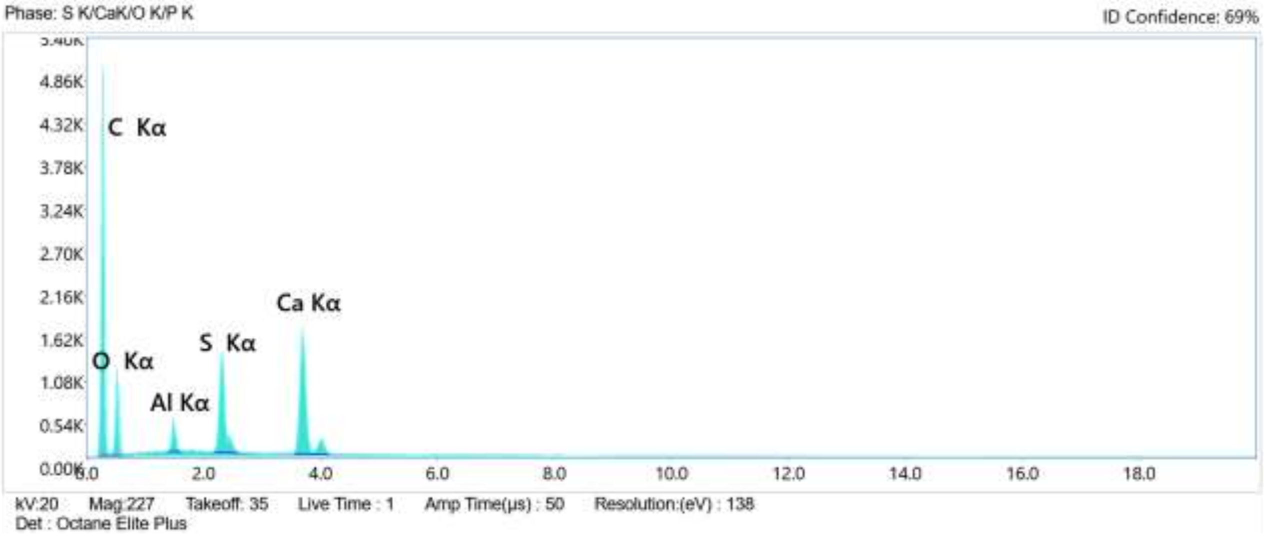

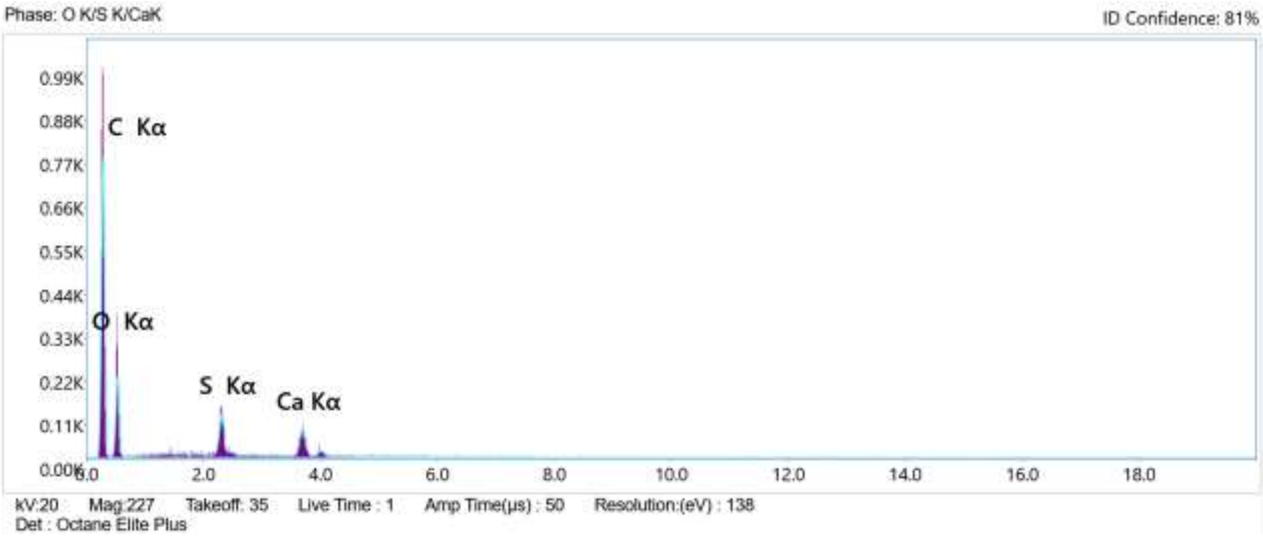

PeBaZAF Quant Result - Analysis Uncertainty: 99.00 %

Element	Weight %	Atomic %	Error %	A
C K	63.82	72.94	11.74	1.0000
O K	27.77	23.83	23.55	1.0000
S K	4.13	1.77	27.96	1.0186
Ca K	4.29	1.47	29.63	1.0075
