# Supplementary material for: Phosphogypsum Processing into Blue Fluorescent Pigments Under Ultraviolet Excitation
Source: Molecules. 2026 Jun 23;31(13):2202. doi: 10.3390/molecules31132202 (PMC13363016; doi:10.3390/molecules31132202)

EDAX APEX

EDAX AMETEK MATERIALS ANALYSIS DIVISION

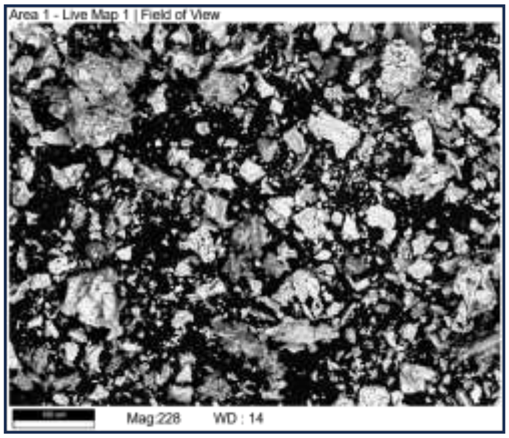

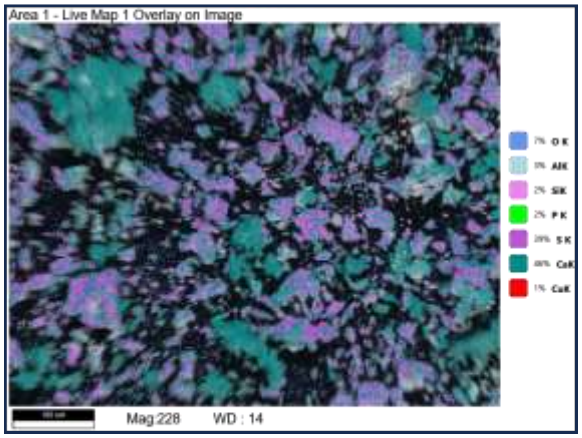

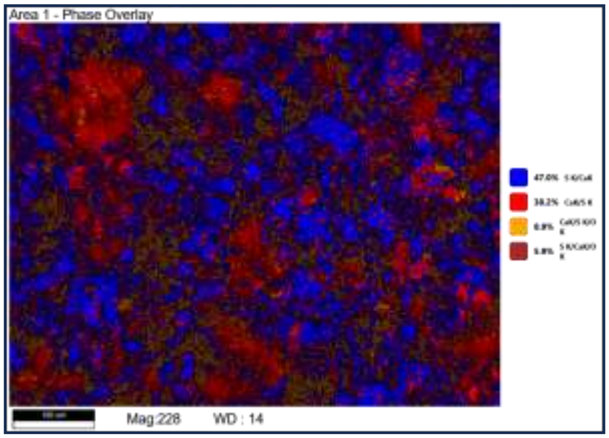

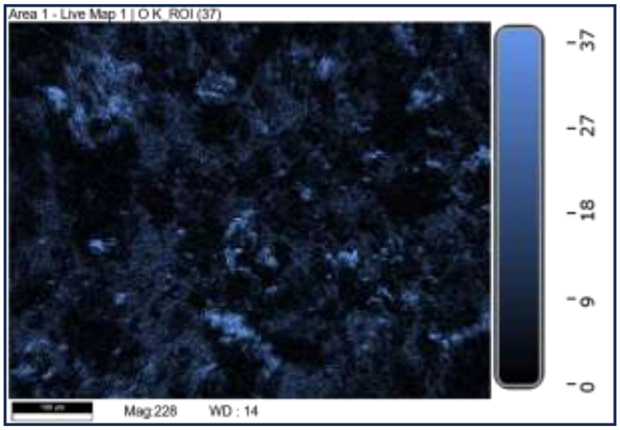

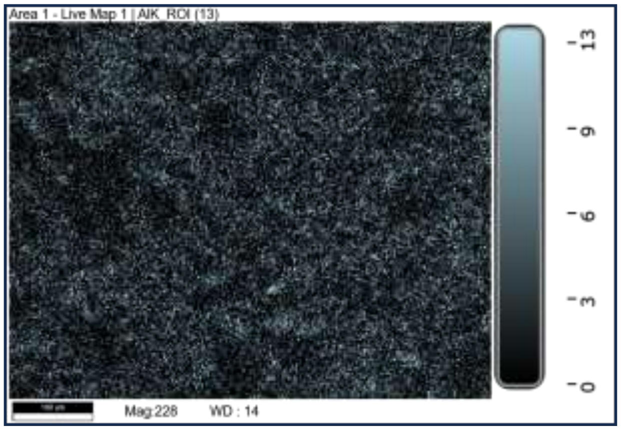

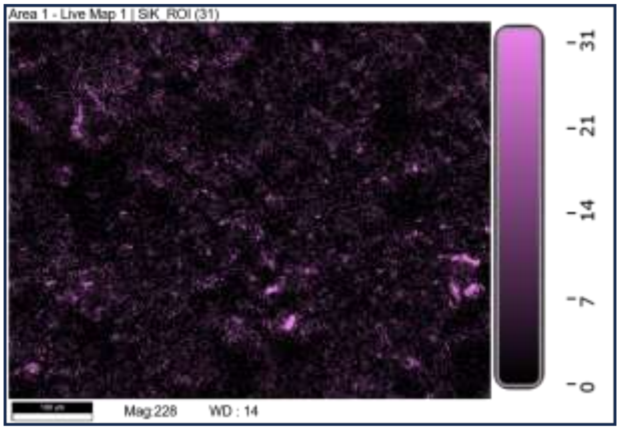

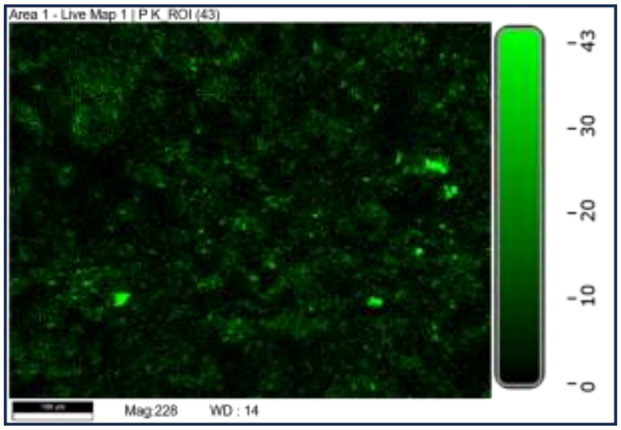
Area 1 - Live Map 1 | P K_ROI (43)
43
30
20
10
0
Mag:228
WD : 14

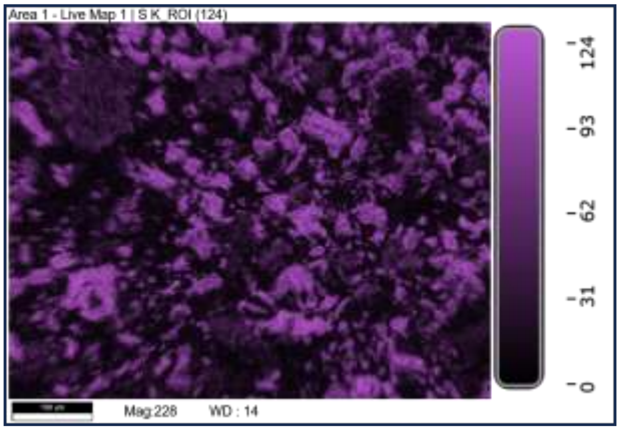
Area 1 - Live Map 1 | S K_ROI (124)
124
93
62
31
0
Mag:228
WD : 14

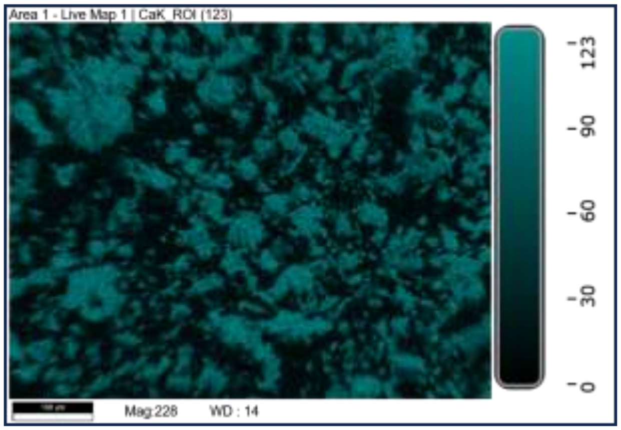
Area 1 - Live Map 1 | CaK_ROI (123)
123
90
60
30
0
Mag:228
WD : 14

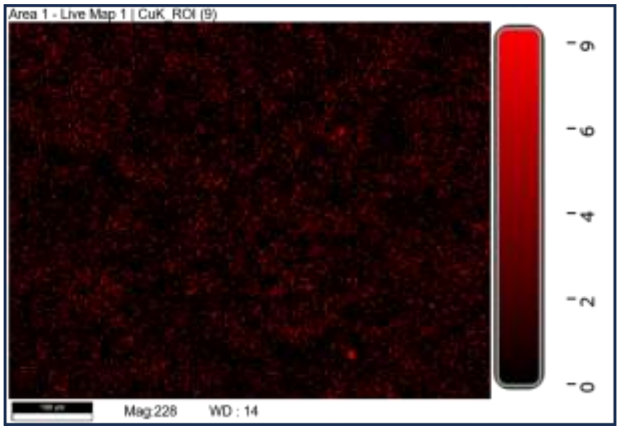
Area 1 - Live Map 1 | CuK_ROI (9)
9
6
4
2
0
Mag:228
WD : 14

EDAX APEX

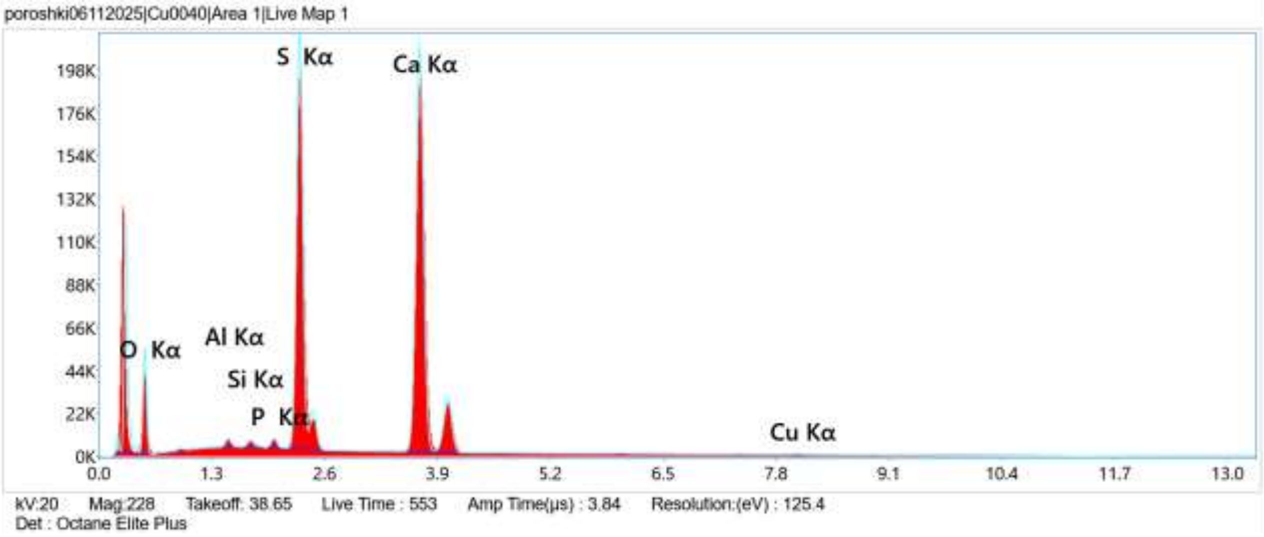

PeBaZAF Quant Result - Analysis Uncertainty: 99.00 %

Element	Weight %	Atomic %	Error %	A
O K	24.84	43.04	15.38	1.0000
Al K	0.43	0.44	8.08	1.0479
Si K	0.35	0.35	8.64	1.0362
P K	0.63	0.57	7.98	1.0272
S K	27.18	23.50	3.64	1.0206
Ca K	46.16	31.93	2.50	1.0147
Cu K	0.40	0.17	39.31	1.0053

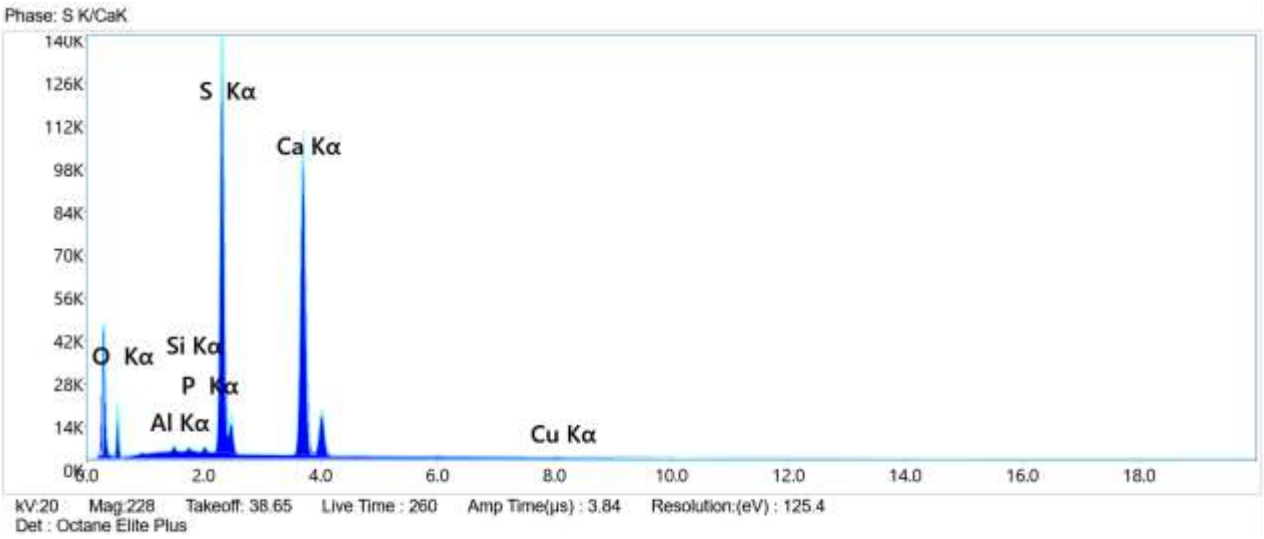

PeBaZAF Quant Result - Analysis Uncertainty: 99.00 %

Element	Weight %	Atomic %	Error %	A
O K	17.57	32.52	15.67	1.0000
Al K	0.35	0.38	11.71	1.0492
Si K	0.26	0.27	16.48	1.0373
P K	0.44	0.42	14.46	1.0280
S K	34.44	31.82	4.51	1.0212
Ca K	46.50	34.37	3.03	1.0177
Cu K	0.45	0.21	41.63	1.0061

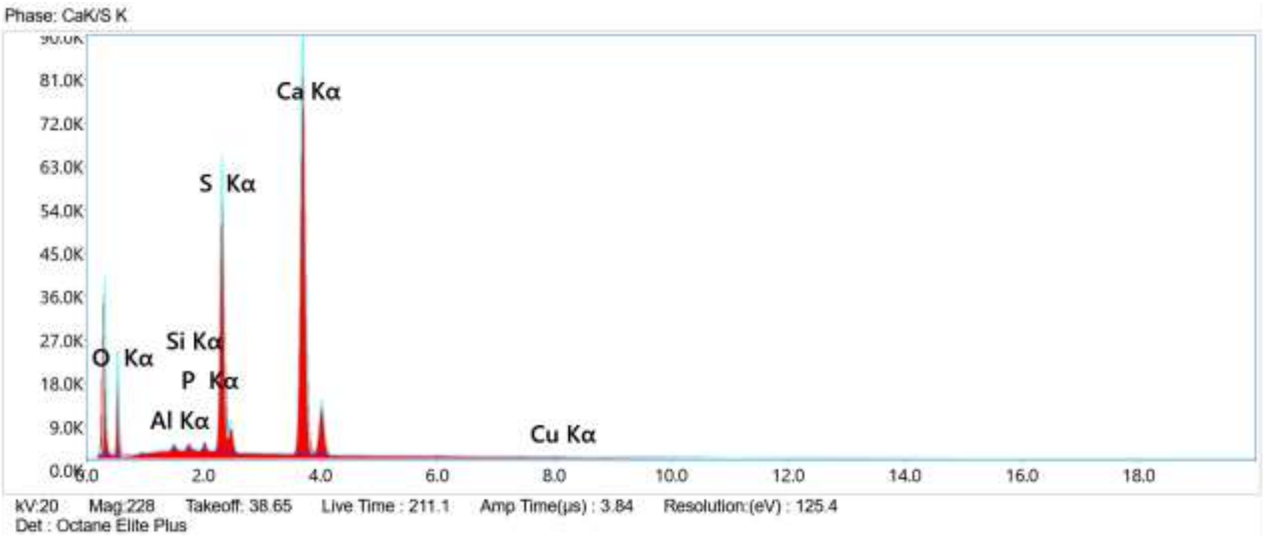

PeBaZAF Quant Result - Analysis Uncertainty: 99.00 %

Element	Weight %	Atomic %	Error %	A
O K	29.98	49.93	15.60	1.0000
Al K	0.35	0.35	13.03	1.0519
Si K	0.34	0.33	14.22	1.0396
P K	0.62	0.54	12.23	1.0299
S K	19.76	16.42	4.48	1.0229
Ca K	48.58	32.29	3.46	1.0139
Cu K	0.36	0.15	68.72	1.0057

EDAX APEX

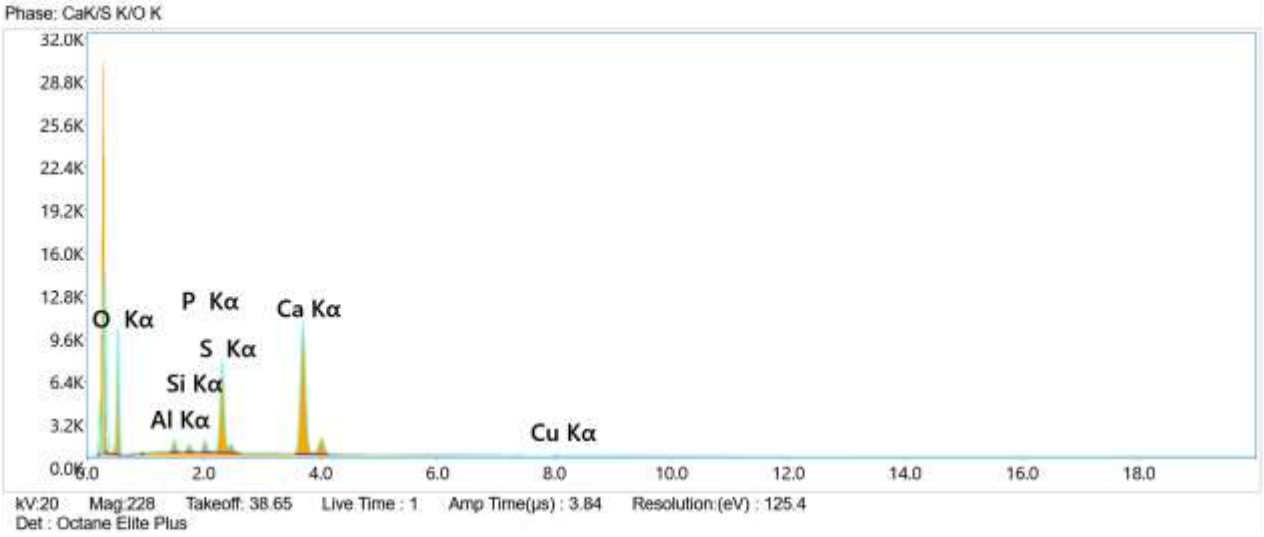

PeBaZAF Quant Result - Analysis Uncertainty: 99.00 %

Element	Weight %	Atomic %	Error %	A
O K	60.85	77.37	16.23	1.0000
Al K	1.57	1.18	15.40	1.0520
Si K	0.62	0.45	24.62	1.0407
P K	0.62	0.41	33.31	1.0311
S K	16.91	10.73	7.28	1.0237
Ca K	19.42	9.86	6.84	1.0133

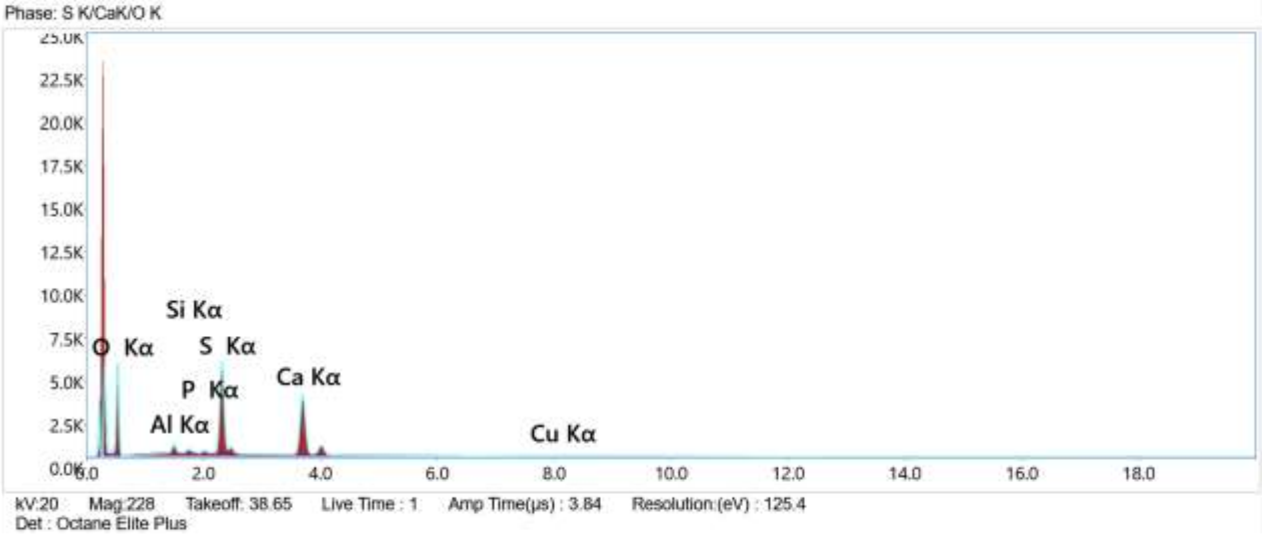

Supplement: Supplementary file 1 [file molecules-31-02202-s001.zip › S 2.pdf]
