# Supplementary material for: Phosphogypsum Processing into Blue Fluorescent Pigments Under Ultraviolet Excitation
Source: Molecules. 2026 Jun 23;31(13):2202. doi: 10.3390/molecules31132202 (PMC13363016; doi:10.3390/molecules31132202)
Supplement: Supplementary file 1 [file molecules-31-02202-s001.zip › S 3.pdf]

EDAX APEX

poroshki_06112025

Author: Apex User

Creation: 11/6/2025 5:39:22 PM

Sample Name: Cu0042

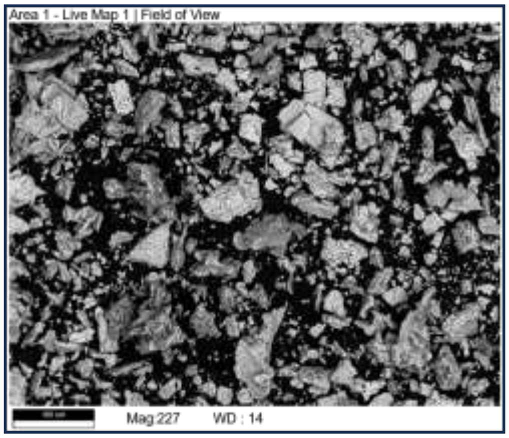

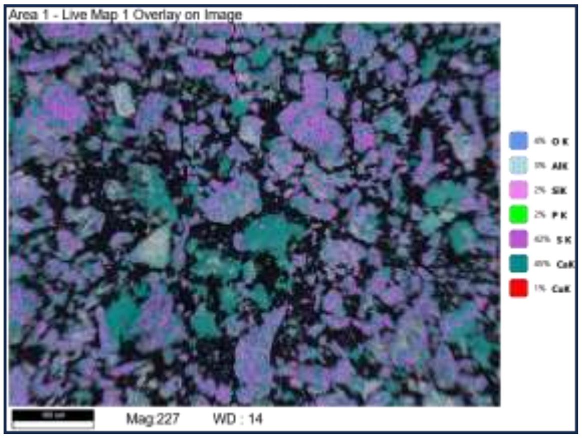

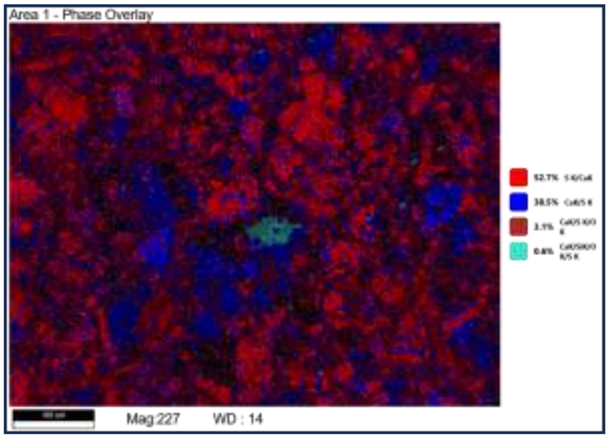

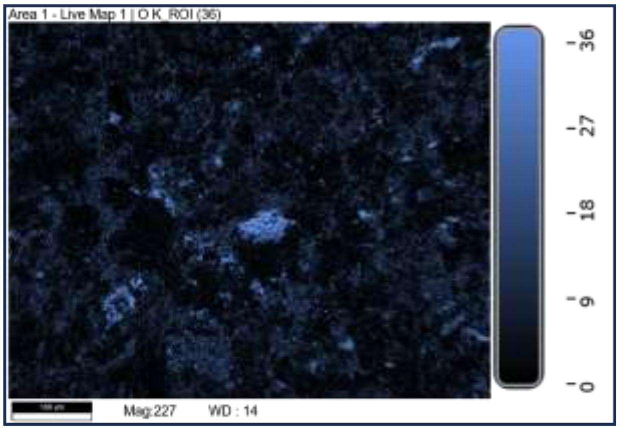

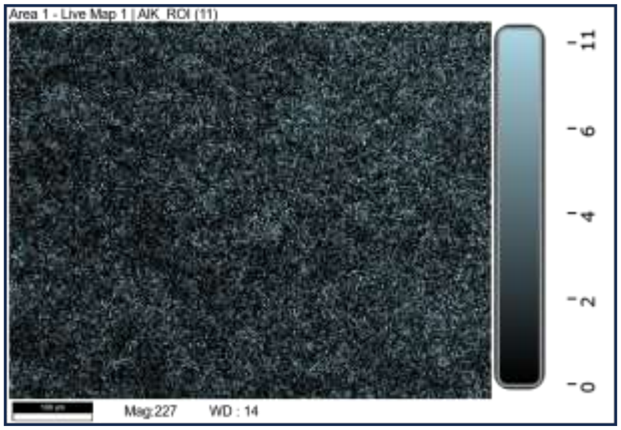

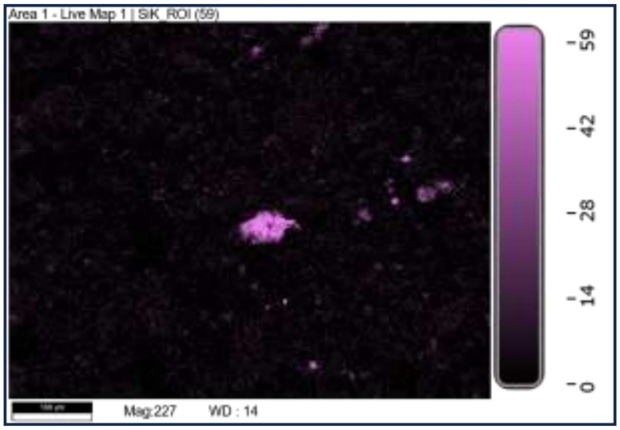

EDAX APEX

poroshki_06112025

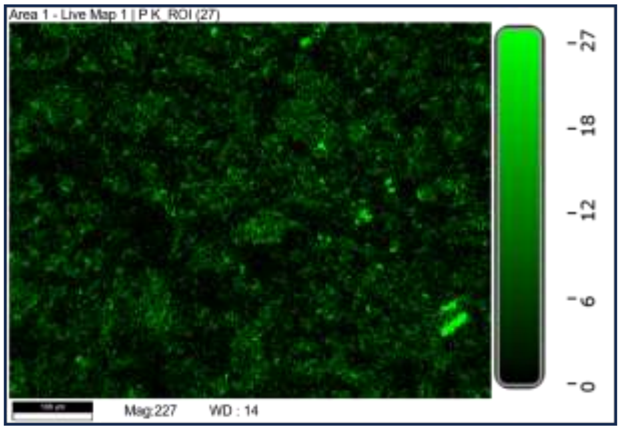

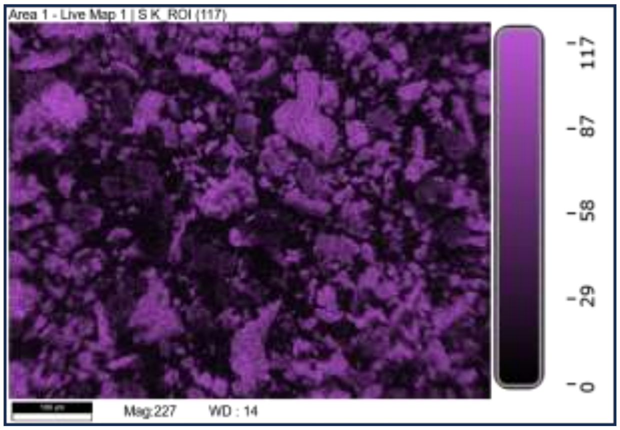

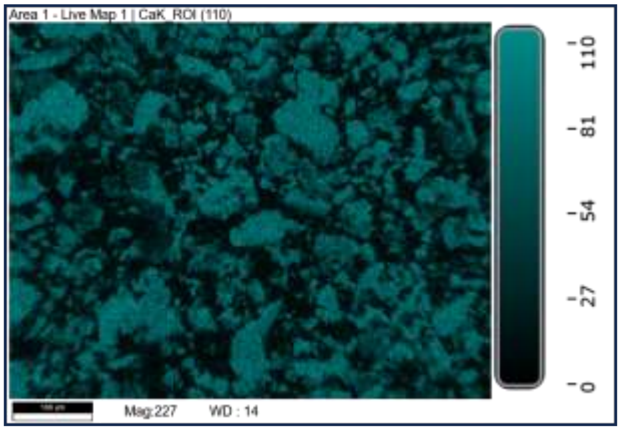

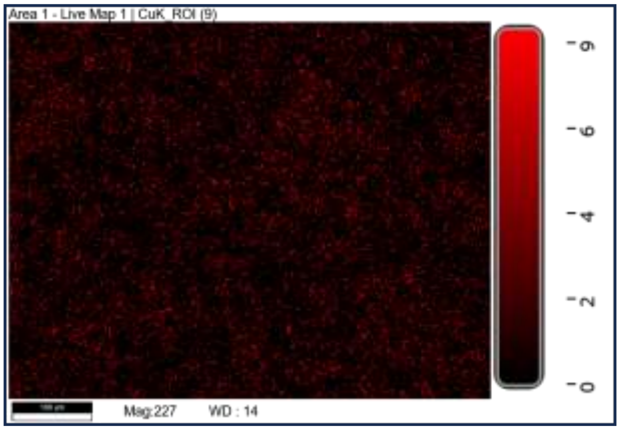

EDAX APEX

poroshki_06112025

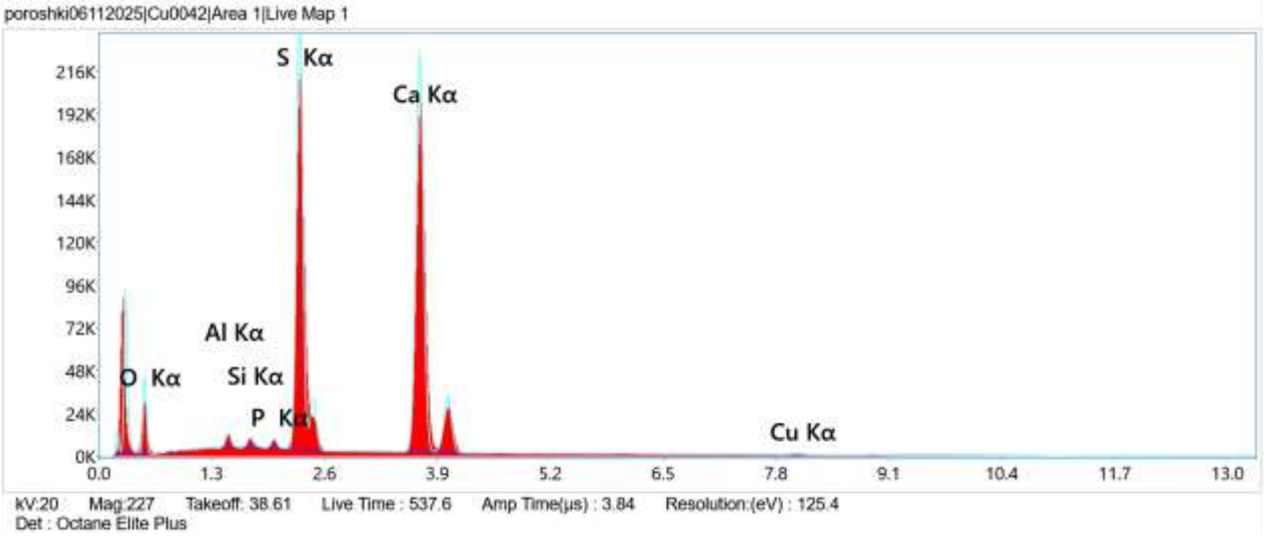

PeBaZAF Quant Result - Analysis Uncertainty: 99.00 %

Element	Weight %	Atomic %	Error %	A
O K	19.02	34.81	15.43	1.0000
Al K	0.75	0.82	6.69	1.0473
Si K	0.52	0.54	7.55	1.0360
P K	0.66	0.62	7.78	1.0272
S K	30.91	28.23	3.77	1.0206
Ca K	47.41	34.64	2.50	1.0158
Cu K	0.74	0.34	15.87	1.0056

EDAX APEX

poroshki_06112025

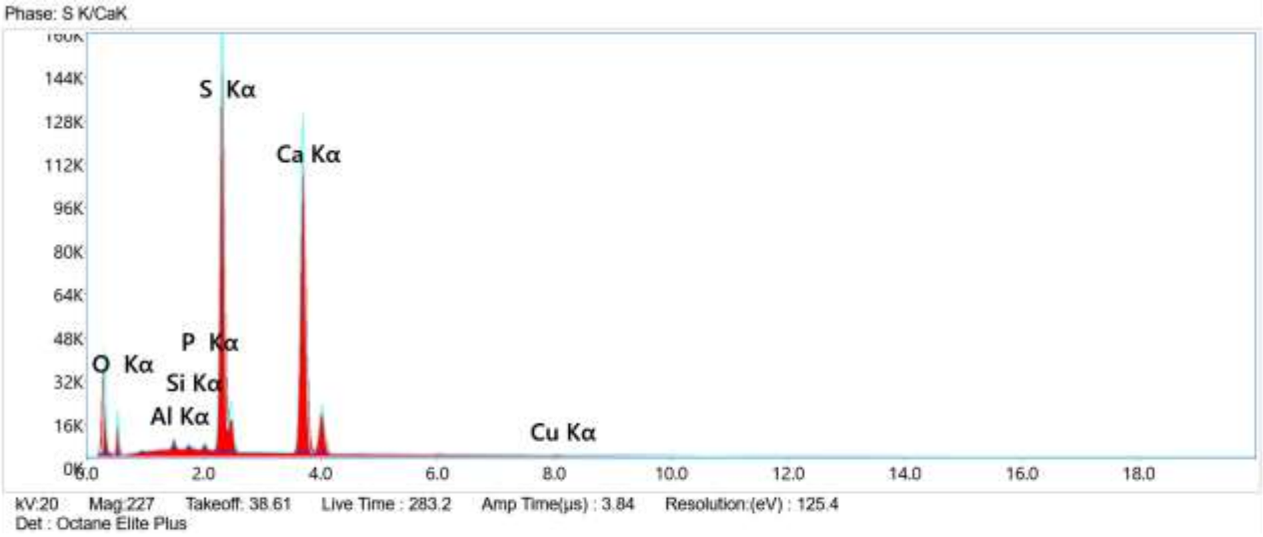

PeBaZAF Quant Result - Analysis Uncertainty: 99.00 %

Element	Weight %	Atomic %	Error %	A
O K	14.67	27.94	15.69	1.0000
Al K	0.66	0.74	8.25	1.0487
Si K	0.29	0.32	13.79	1.0372
P K	0.52	0.51	13.83	1.0279
S K	36.10	34.30	4.49	1.0212
Ca K	47.37	36.01	2.92	1.0182
Cu K	0.38	0.18	45.74	1.0062

EDAX APEX

poroshki_06112025

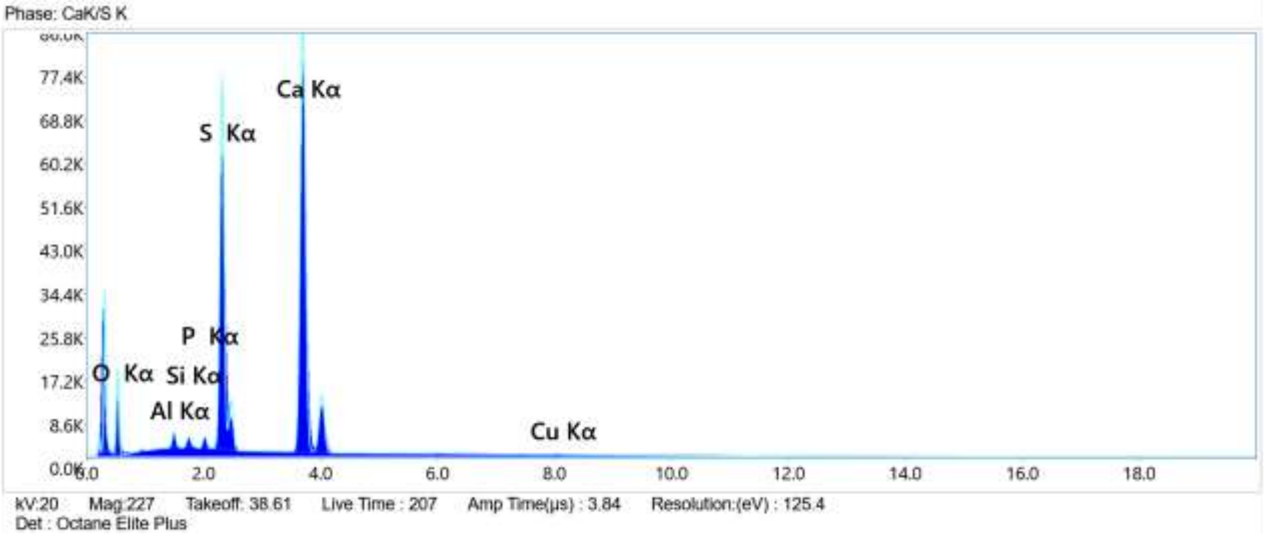

PeBaZAF Quant Result - Analysis Uncertainty: 99.00 %

Element	Weight %	Atomic %	Error %	A
O K	47.26	67.19	16.54	1.0000
Al K	2.22	1.87	16.52	1.0526
Si K	0.94	0.76	26.84	1.0418
P K	1.22	0.90	26.01	1.0322
S K	14.07	9.98	10.76	1.0251
Ca K	33.50	19.01	9.57	1.0129
Cu K	0.78	0.28	100.00	1.0044

EDAX APEX

poroshki_06112025

Phase: CaK/S K/O K

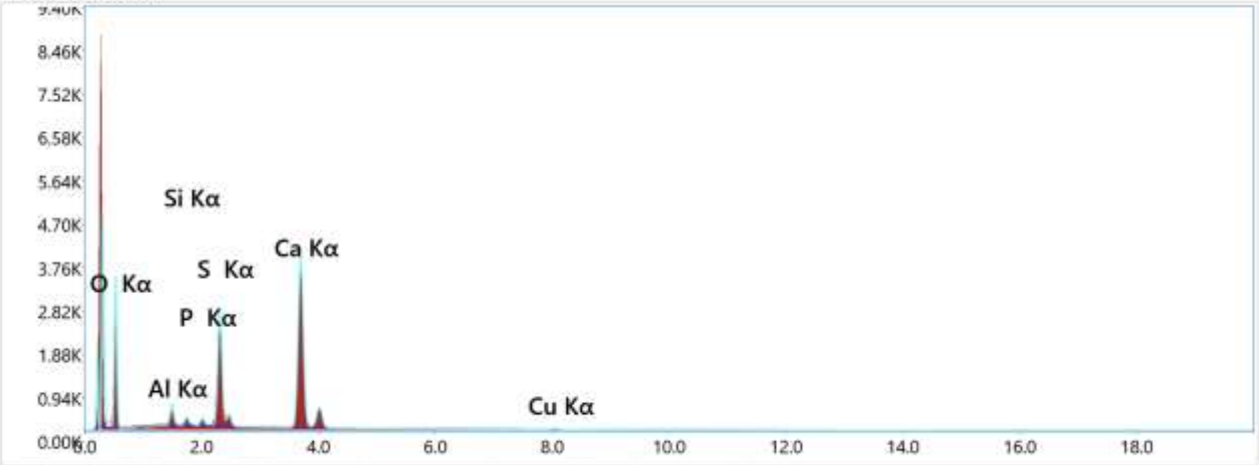

kV:20 Mag:227 Takeoff: 38.61 Live Time : 1 Amp Time(µs) : 3.84 Resolution:(eV) : 125.4
Det : Octane Elite Plus

EDAX APEX

poroshki_06112025

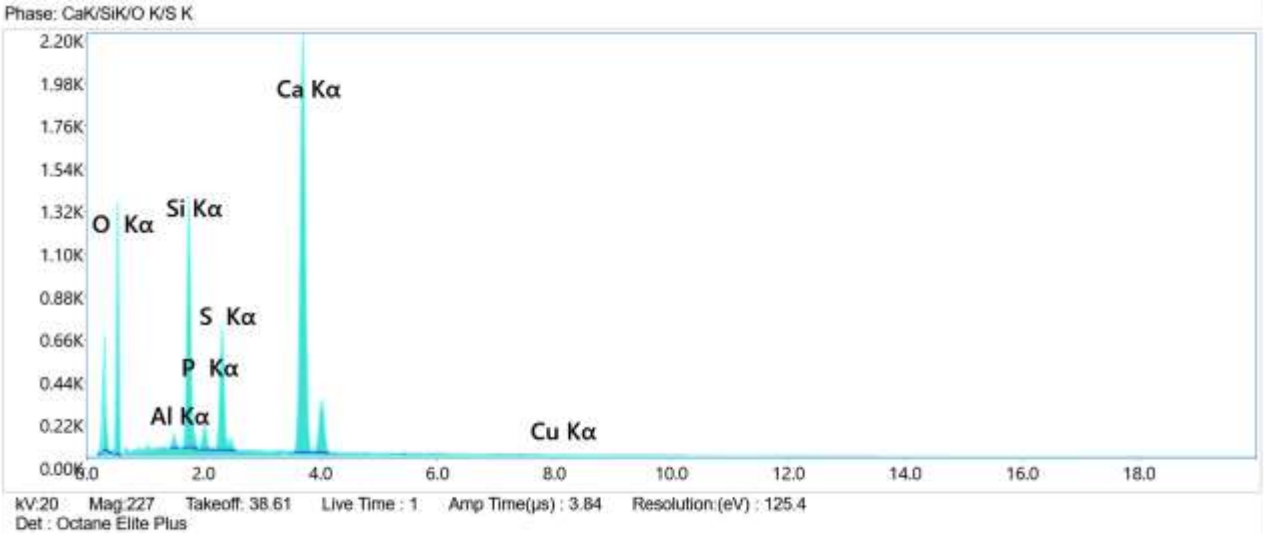

PeBaZAF Quant Result - Analysis Uncertainty: 99.00 %

Element	Weight %	Atomic %	Error %	A
O K	38.61	58.42	17.45	1.0000
Al K	0.67	0.60	44.86	1.0510
Si K	11.19	9.65	14.37	1.0391
P K	1.64	1.28	35.11	1.0380
S K	7.45	5.62	15.16	1.0301
Ca K	40.43	24.42	11.95	1.0126
