# Supplementary material for: Phosphogypsum Processing into Blue Fluorescent Pigments Under Ultraviolet Excitation
Source: Molecules. 2026 Jun 23;31(13):2202. doi: 10.3390/molecules31132202 (PMC13363016; doi:10.3390/molecules31132202)
Supplement: Supplementary file 1 [file molecules-31-02202-s001.zip › S 4.pdf]

Table. Spectrum decomposition parameters C1s, O1s, S2p, Ca2p и Cu2p.

Connection	Communication energy, eV	FWHM, eV	The area under the peak	The share of communication, %
Sample Cu 0.4 % mol.				
C-C sp^2	284,6	1,85	14568,0	55,64
C-O(-OH)	285,89	1,85	2039,8	7,79
OH-C=O, C-CO_3	289,38	1,85	9577,1	36,57
O in $(CO_3)^{2-}$	531,11	2,14	56186,6	26,09
O in $(SO_4)^{2-}$	531,86	2,14	159177,5	73,91
$S2p_{3/2}$ in SO_4^{2-}	168,79	1,65	11169,8	30,17
$S2p_{1/2}$ in SO_4^{2-}	170,04	1,59	5584,9	15,08
$S2p_{3/2}$ in S^{2-}	160,05	1,62	12830,3	34,65
$S2p_{1/2}$ in S^{2-}	161,35	1,48	4520,3	12,21
S2p sat in $CaSO_4$	166,86	1,72	2921,2	7,89
$Ca2p_{3/2}$ in CaS	345,95	1,74	43734,3	23,67
$Ca2p_{1/2}$ in CaS	348,67	2,27	22006,5	11,91
$Ca2p_{3/2}$ in $CaSO_4$	347,35	1,74	68908,9	37,30
$Ca2p_{1/2}$ in $CaSO_4$	350,81	2,27	50085,1	27,11
$Cu2p_{3/2}$	932,01	2,06	2591,2	71,79
$Cu2p_{1/2}$	951,69	2,03	1018,1	28,21
Sample Cu 0.6 % mol.				
C-C sp^2	284,6	1,84	16229,5	53,72
C-O(-OH)	286,38	1,84	2864,0	8,48
OH-C=O, C-CO_3	289,14	1,84	11115,9	36,80
O in $(CO_3)^{2-}$	531,31	2,19	150045,6	70,41
O in $(SO_4)^{2-}$	532,59	2,19	63064,6	29,59
$S2p_{3/2}$ in SO_4^{2-}	168,62	1,65	9995,5	27,39
$S2p_{1/2}$ in SO_4^{2-}	169,87	1,59	4997,8	13,70
$S2p_{3/2}$ in S^{2-}	159,90	1,62	13267,5	36,36
$S2p_{1/2}$ in S^{2-}	161,21	1,56	5556,5	15,23
S2p sat in $CaSO_4$	166,72	1,67	2672,1	7,32
$Ca2p_{3/2}$ in CaS	345,81	1,73	47135,5	27,24
$Ca2p_{1/2}$ in CaS	348,55	2,40	23618,8	13,65
$Ca2p_{3/2}$ in $CaSO_4$	347,17	1,73	57129,6	33,01
$Ca2p_{1/2}$ in $CaSO_4$	350,59	2,40	45178,8	26,11
$Cu2p_{3/2}$	931,67	1,96	4309,0	72,00
$Cu2p_{1/2}$	951,45	2,15	1675,7	28,00

Connection	Communication energy, eV	FWHM, eV	The area under the peak	The share of communication, %
Sample Cu 0.8 % mol.				
C-C sp^2	284,6	1,80	16192,8	53,61
C-O(-OH)	286,30	1,80	3010,0	9,97
OH-C=O, C-CO_3	289,21	1,80	11000,4	36,42
O in $(CO_3)^{2-}$	531,24	2,08	134394,9	66,22
O in $(SO_4)^{2-}$	531,99	2,08	62367,7	30,73
H_2O	533,82	2,08	6189,2	3,05
$S2p_{3/2}$ in SO_4^{2-}	168,43	1,78	7901,3	23,05
$S2p_{1/2}$ in SO_4^{2-}	169,68	1,66	3950,6	11,52
$S2p_{3/2}$ in S^{2-}	159,93	1,53	12181,1	35,54
$S2p_{1/2}$ in S^{2-}	161,19	1,74	7195,6	20,99
S2p sat in $CaSO_4$	166,56	1,58	3049,7	8,90
$Ca2p_{3/2}$ in $CaSO_4$	347,06	1,46	30011	26,98687749
$Ca2p_{1/2}$ in $CaSO_4$	350,54	2,16	21272,8	19,12920088
$Ca2p_{3/2}$ in CaS	345,7	1,46	38684,3	34,7861939
$Ca2p_{1/2}$ in CaS	348,91	2,16	21237,8	19,09772773
$Cu2p_{3/2}$	931,67	1,96	4309,0	72,00
$Cu2p_{1/2}$	951,45	2,15	1675,7	28,00
